# Artificial Intelligence Analysis of Gene Expression Predicted the Overall Survival of Mantle Cell Lymphoma and a Large Pan-Cancer Series

**DOI:** 10.3390/healthcare10010155

**Published:** 2022-01-14

**Authors:** Joaquim Carreras, Naoya Nakamura, Rifat Hamoudi

**Affiliations:** 1 Department of Pathology, Faculty of Medicine, Tokai University School of Medicine, 143 Shimokasuya, Isehara 259-1193, Japan; naoya@is.icc.u-tokai.ac.jp; 2 Department of Clinical Sciences, College of Medicine, University of Sharjah, Sharjah P.O. Box 27272, United Arab Emirates; rhamoudi@sharjah.ac.ae; 3 Division of Surgery and Interventional Science, University College London, Gower Street, London WC1E 6BT, UK

**Keywords:** mantle cell lymphoma, gene expression, MCL35 assay, artificial intelligence, machine learning, deep learning, artificial neural network, multilayer perceptron, immuno-oncology, overall survival

## Abstract

Mantle cell lymphoma (MCL) is a subtype of mature B-cell non-Hodgkin lymphoma characterized by a poor prognosis. First, we analyzed a series of 123 cases (GSE93291). An algorithm using multilayer perceptron artificial neural network, radial basis function, gene set enrichment analysis (GSEA), and conventional statistics, correlated 20,862 genes with 28 MCL prognostic genes for dimensionality reduction, to predict the patients’ overall survival and highlight new markers. As a result, 58 genes predicted survival with high accuracy (area under the curve = 0.9). Further reduction identified 10 genes: *KIF18A*, *YBX3*, *PEMT*, *GCNA*, and *POGLUT3* that associated with a poor survival; and *SELENOP*, *AMOTL2*, *IGFBP7*, *KCTD12*, and *ADGRG2* with a favorable survival. Correlation with the proliferation index (Ki67) was also made. Interestingly, these genes, which were related to cell cycle, apoptosis, and metabolism, also predicted the survival of diffuse large B-cell lymphoma (GSE10846, *n* = 414), and a pan-cancer series of The Cancer Genome Atlas (TCGA, *n* = 7289), which included the most relevant cancers (lung, breast, colorectal, prostate, stomach, liver, etcetera). Secondly, survival was predicted using 10 oncology panels (transcriptome, cancer progression and pathways, metabolic pathways, immuno-oncology, and host response), and *TYMS* was highlighted. Finally, using machine learning, C5 tree and Bayesian network had the highest accuracy for prediction and correlation with the LLMPP MCL35 proliferation assay and RGS1 was made. In conclusion, artificial intelligence analysis predicted the overall survival of MCL with high accuracy, and highlighted genes that predicted the survival of a large pan-cancer series.

## 1. Introduction

Mantle cell lymphoma (MCL) is a hematological neoplasia derived from B-lymphocytes, and a subtype of non-Hodgkin lymphomas (NHL) [1]. MCL represents around 7% of adult NHL, and has an incidence of four to eight cases per million people per year [2,3,4,5,6]. MCL affects white men, with a median age at diagnosis of 65 years. The disease frequency increases with age [7], and the incidence of this disease is on the rise in Western and developed countries [7].

MCL is a B-cell lymphoma of small and irregular cells (centrocytes) [8]. The immunophenotype of the classic variant is characterized by the expression of B-cell markers (CD19, CD20), CD5, SOX11, and cyclin D1 due to the characteristics translocation t(11; 14) (q13; q32) between *CCND1* and *IGH* locus [9,10,11]. MCL expresses high levels of IgM and IgD, with a lambda light chain restriction in 80% of the cases [8,12]. At diagnosis, most of the patients present with an advanced disease, and lymphadenopathy. Primary extranodal disease is found in 20% of cases, and the gastrointestinal site in the form of lymphomatous polyposis is a characteristic location [13,14,15].

MCL has traditionally been considered a very aggressive and incurable lymphoma. MCL is associated with a median survival of 3–5 years, with most patients not being cured even with the newer therapeutic modalities [1,8,16]. The “leukemic” variant, which is SOX11-negative, is clinically indolent [17]. Several studies have focused on the identification of prognostic markers to identify patients with a higher probability of an aggressive disease [18,19,20,21,22,23,24,25,26,27]. Among them, the International Prognostic Index (IPI), MCL International Prognostic Index (MIPI), and proliferation index (Ki67) are extensively used [18,22]. The pathobiology of MCL comprises several pathways, mechanisms, and target genes that contribute to not only in the pathogenesis but also to aggressiveness and clinical evolution. The major oncogenic driver is *CCND1* gene of the cell cycle pathway. Other relevant genes are involved in cell cycle (*CCND2*, *CCND3*, *MYC*), response to DNA damage (*ATM*, *TP53*), chromatin modification (*WHSC1*, *MLL2*, *MEF2B*), apoptosis (BCL2, *BIRC3*, *TLR2*), and NOTCH signaling (*NOTCH1* and *NOTCH2*), NF-kB and PI3K/AKT signaling pathways, among others [8,28,29,30,31].

Neural networks are a favored analytical method for numerous predictive data mining applications because of their power, adaptability, and ease of usage. Predictive neural networks are specially valuable in applications where the underlying process is complex [32,33,34,35,36,37,38,39,40,41,42,43], such as biological systems [44]. Both the multilayer perceptron (MLP) and radial basis function (RBF) network have a feedforward architecture, because the connections in the network flow forward the input layer (predictors) to the output layer (responses). The hidden layer contains unobservable nodes or units. The value of each hidden unit is some function of the predictors. Both are supervised learning networks that perform prediction and classification. Your choice of strategy will depend on the sort of data and the level of complexity you look for to reveal; while the MLP strategy can discover more complex connections, the RBF method is faster [32,33]. We have recently shown that neural networks can predict the prognosis of diffuse large B-cell lymphoma (DLBCL) and follicular lymphoma (FL) [35,37,45], and also can predict the different subtypes of non-Hodgkin lymphomas with high accuracy [46]. In this research we focused on MCL and the workflow algorithm was improved to handle this type of lymphoma more efficiently: the neural networks not only predicted the overall survival outcome and identified the most relevant genes, but the results were modulated by the inclusion of known prognostic genes and immune oncology pathways.

The main aim of the work was to use artificial neural networks (ANN) analyses and other machine learning techniques to analyze the gene expression of MCL and identify relevant prognostic markers. The principal conclusion was that ANN provided a novel analysis technique that not only confirmed known prognostic markers but also highlighted new potential pathological mechanisms.

## 2. Materials and Methods

### 2.1. Hardware

All the analyses were performed on a desktop workstation using an AMD Ryzen 7, 3700X, 8-core, processor at 2.59 GHz, 16.0 GB RAM, and a Nvidia GeForce GTX 1650 Turing architecture, 4 GB, GPU.

### 2.2. Software

Several software were used for data processing, preanalysis, full-analysis, and validation including EditPad Lite, Microsoft Excel, R, R Studio, IBM SPSS Statistic and Modeler, GSEA, and JMP.

The details of the software were as follows:

- EditPad Lite 8 (Just Great Software Co. Ltd., Rawai Phuket 83130, Thailand; page URL: http://www.just-great-software.com/aboutjg.html (accessed on 29 August 2021));
- Microsoft Excel 2016 [(16.0.5173.1000) MSO (16.0.5173.1000) 64-bit, Microsoft K.K., Shinagawa, Tokyo, Japan; page URL: https://www.microsoft.com/ja-jp/microsoft-365/excel (accessed on 29 August 2021)];
- R 3.6.3 (page URL: https://www.r-project.org/ (accessed on 29 August 2021) [47]);
- R Studio 1.3.959 (R Studio, Boston, MA 02210, USA; page URL: https://www.rstudio.com/products/rstudio/#rstudio-desktop (accessed on 29 August 2021));
- IBM SPSS Statistics 26 and Modeler 18 (IBM Japan Ltd., Tokyo 103-8510, Japan; page URL: https://www.ibm.com/jp-ja/analytics/spss-statistics-software (accessed on 29 August 2021));
- Gene Set Enrichment Analysis (GSEA) 4.1.0 (UC San Diego, Broad Institute, Cambridge, MA 02142, USA; page URL: http://www.gsea-msigdb.org/gsea/index.jsp (accessed on 29 August 2021) [48,49]); https://github.com/GSEA-MSigDB/gsea-desktop (accessed on 8 December 2021);
- JMP Pro 14 Statistical Discovery (SAS Institute Inc., Cary, NC 27513-2414, USA; page URL: https://www.jmp.com/ja_jp/home.html (accessed on 29 August 2021));
- Morpheus matrix visualization and analysis software (Broad Institute, Cambridge, MA 02142, USA), https://software.broadinstitute.org/morpheus) (accessed on 29 November 2021);
- String (version 11, String consortium 2020) [19]; https://string-db.org/ (accessed on 29 November 2021).

### 2.3. Predictive Genes and Artificial Neural Network Analysis

#### 2.3.1. Gene Expression Series of Mantle Cell Lymphoma

The gene expression data of the MCL series GSE93291 were downloaded from the gene expression omnibus (GEO) database [50], which is located at the National Center for Biotechnology Information (NCBI) repository [page URL: https://www.ncbi.nlm.nih.gov/ (accessed on 29 August 2021)]. This database was last updated on 25 March 2019 (contact name: Professor Louis M. Staudt, National Cancer Institute, Lymphoid Malignancies Branch laboratory, Bethesda, MD 20892, USA). 

The study involved retrospective gene expression profiling of samples from patients with MCL, confirmed by expert pathology consensus review. This series was created by the Lymphoma/Leukemia Molecular Profiling Project (LLMPP) [50]. These biopsies, with tumor content ≥ 60%, were obtained from untreated patients, with no history of previous lymphoma, who subsequently received a broad range of treatment regimens. The biopsies contributing to the set included 80 biopsies described in Rosenwald et al. [51] (classified based on established morphologic and immunophenotypic criteria, with overexpression of cyclin D1 (*CCND1*) mRNA (in most cases, immunohistochemistry demonstrated overexpression of cyclin D1 also on the protein level), 3.8 male/female ratio, median age of 62 years (range 38 to 93), multiagent treatment, and median survival 2.8 years) [51], along with additional biopsies gathered from the clinical sites of the LLMPP. The treatments of the patients was multiagent chemotherapy (R-CHOP, R-CHOP-like), six received no treatment, and no information on treatment was available for two patients.

The gene expression array used in this series was the HG-U133 plus 2 platform (GPL570, Affymetrix, Santa Clara, CA, USA). The GeneChip™ Human Genome U133 Plus 2.0 Array (#900466, ThermoFisher Scientific, Affymetrix Japan K.K., Tokyo, Japan), which is the first and most comprehensive whole human genome array. It has a complete coverage of the Human Genome U133 Set, plus 6500 additional genes for analysis of over 47,000 transcripts. The design and performance of the chip can be accessed at the following webpage: https://www.thermofisher.com/order/catalog/product/900466 (accessed on 29 December 2021).

Total RNA from MCL specimens of frozen samples from 123 patients had been extracted using the FastTrack kit from Invitrogen (Thermo Fisher Scientific Corp., Waltham, MA 02451, USA), and biotinylated cRNA had been prepared according to the standard Affymetrix protocol from 1 microg mRNA (Expression Analysis Technical Manual, 2001, Affymetrix). The Affymetrix hybridization protocol was used: following fragmentation, 15 micrograms of cRNA were hybridized for 16 h at 45 °C on arrays from Affymetrix. Arrays were washed and stained in the Affymetrix Fluidics Station 400. The Affymetrix scanning protocol was used and the scanning had been performed by the Affymetrix 3000 scanner. The data had been analyzed with Microarray Suite version 5.0 (MA S 5.0) using Affymetrix default analysis settings and global scaling as normalization method. The trimmed mean target intensity of each array was arbitrarily set to 500. The data was normalized and log2 transformed. The original series matrix files [50] provided by the LLMPP were used for the artificial neural network analysis. The gene expression values were collapsed to symbols applying the max probe values, using the GSEA software and the gene cluster text file (*.gct) [52,53].

#### 2.3.2. Identification of Prognostic Genes for Overall Survival

Eighty-six prognostic and pathogenic genes specific for mantle cell lymphoma (MCL) were selected from previous publications [1,8,17,22,28,29,30,31,50].

Among these 86 genes, 28 genes with prognostic value for overall survival in this GSE93291 series were selected. The selection depended on the presence of a significant *p* value in the Kaplan–Meier with log-rank test, after finding adequate cut-off for the stratification into low vs. high groups (Table 1).

The cut-offs were found using SPSS software on the collapsed to symbols gene expression values dataset (i.e., each gene had only one expression value). The visual binning function created new variables based on grouping contiguous values into a limited number of distinct categories. The cutpoints were created using equal percentiles, three cutpoints and a width of 25%. After visualization of the overall survival plots with the Kaplan–Meier and log-rank test, the most adequate cut-off value was identified. Then, the Cox regression calculated the hazard-risk (contrast: indicator; reference category: first). Based on the p values (Table 2), the most relevant predictors for overall survival were *MKI67* (*p* = 6.6 × 10^−9^, hazard risk = 4.4), *CDK4* (*p* = 3.2 × 10^−8^; HR = 4.0), *CHEK1* (*p* = 0.2 × 10^−5^, HR = 3.0), *CCND1* (*p* = 0.4 × 10^−5^, HR = 3.1), and *CDKN2C* (*p* = 0.8 × 10^−5^, HR = 2.8). These genes belonged to the cell cycle and apoptosis pathways.

#### 2.3.3. Description of the Basic Neural Network Architecture

**Figure 1:**
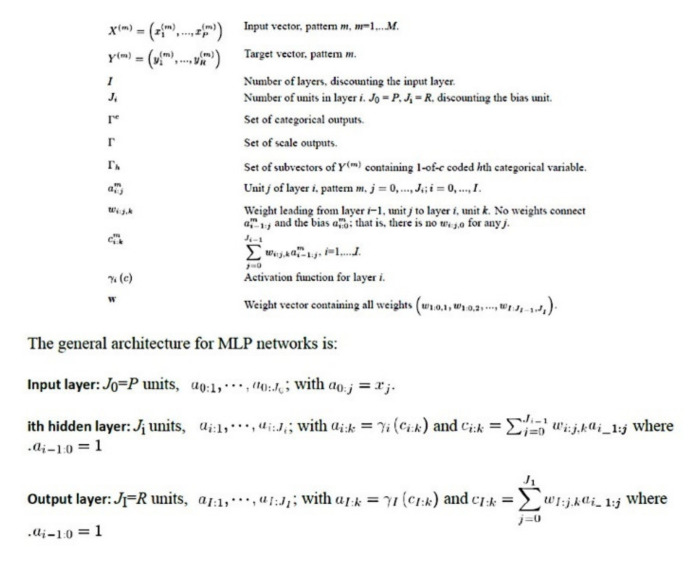
General architecture for multilayer perceptron (MLP) networks. A neural network is a set of non-linear data modeling tools consisting of input layers plus one or two hidden layers. The multilayer perceptron procedure is a feedforward architecture. In comparison to RBF, the MLP con find more complex relationships but it is slower to compute. The MLP network is a function of one or more predictors (also called inputs or independent variables) that minimizes the prediction error of one or more target variables (also called outputs) [32,33,60].

**Figure 2:**
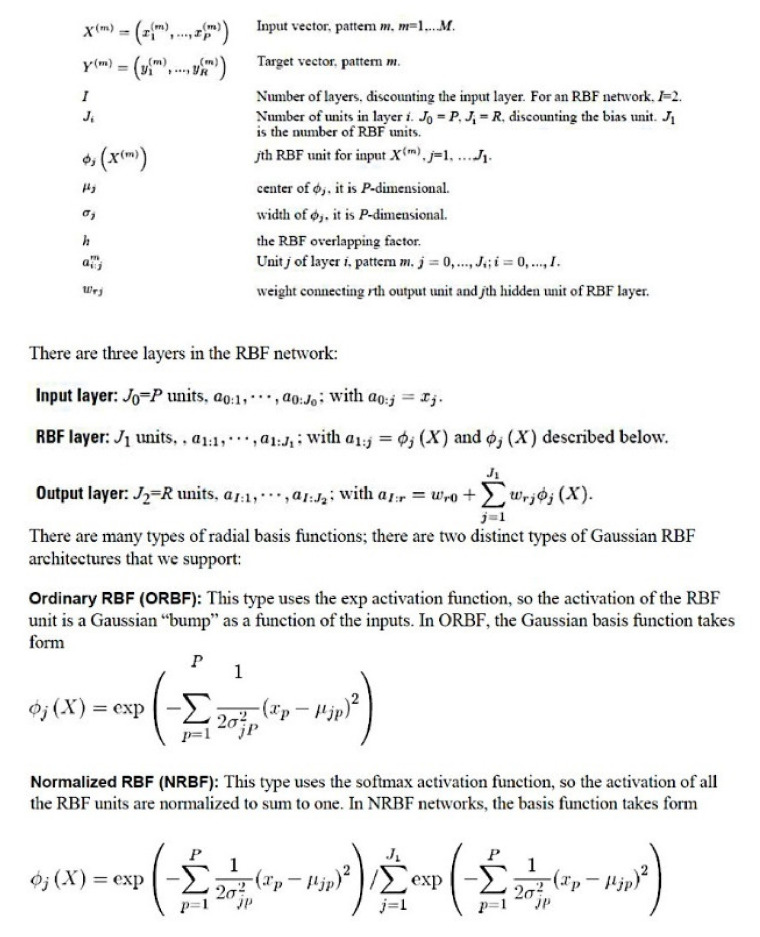
General architecture for radial basis function (RBF) networks. A radial basis function (RBF) network is a feed-forward, supervised learning network with only one hidden layer, called the radial basis function layer [32,33,60].

**Figure 3:**
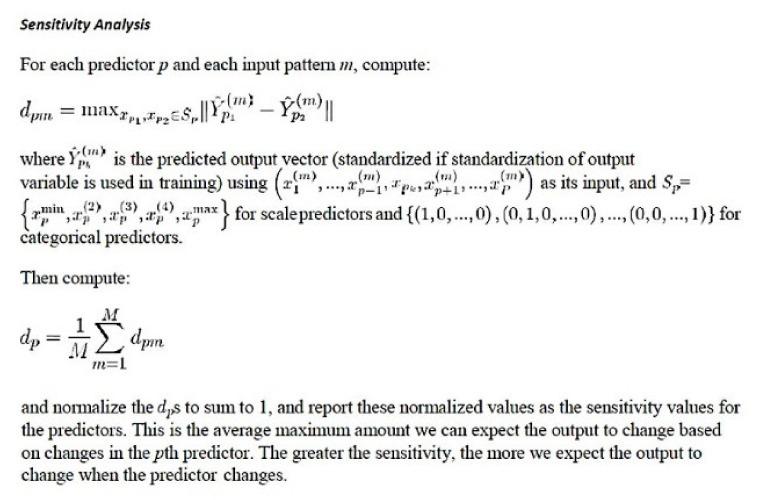
Sensitivity analysis. Independent variable importance analysis. Performs a sensitivity analysis, which computes the importance of each predictor in determining the neural network [32,33,60].

**Figure 4:**
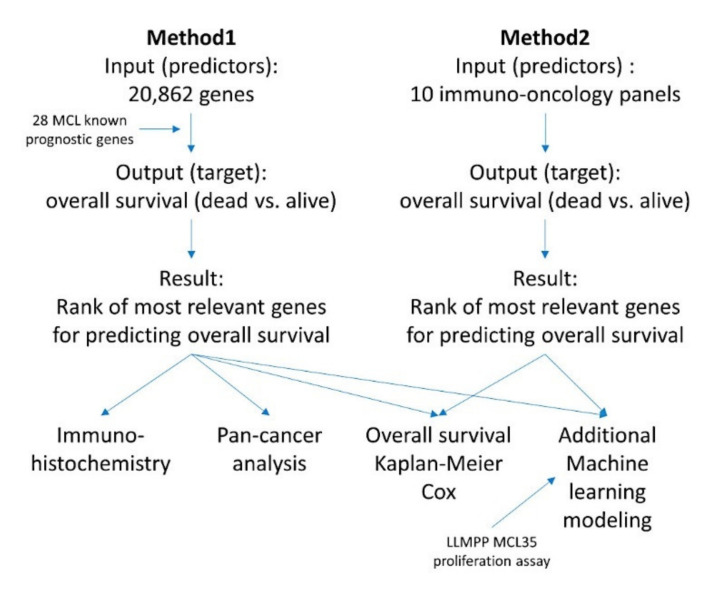
Summary of the analysis methodology. The analysis was comprised of two methods, one based on the analysis of 20,862 genes and a second based on 10 immuno-oncology panels. This research used artificial neural networks and several machine learning techniques to identify genes associated with the overall survival of the patients. Correlation with known MCL pathogenic genes and the LLMPP MCL35 proliferation assay was also made.

**Figure 5:**
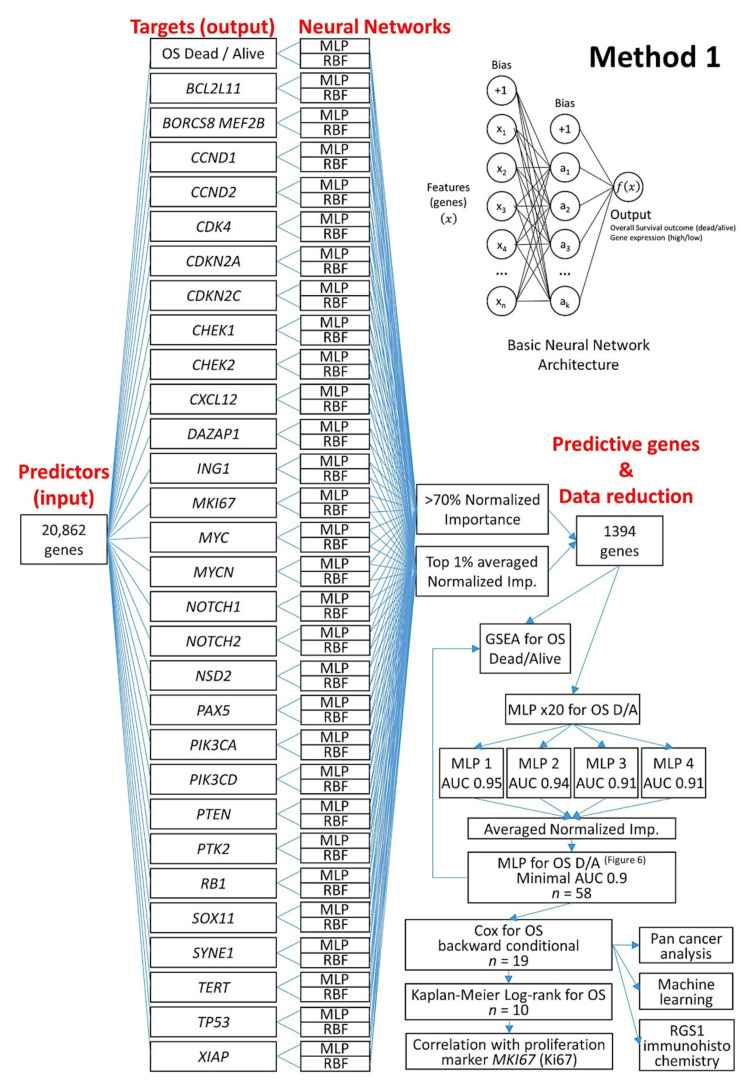
Artificial neural network analysis for the prediction of the overall survival of mantle cell lymphoma (Method 1). From a start point of 20,862 genes, using several neural networks, a correlation between the overall survival outcome and several mantle cell lymphoma pathogenic genes managed to reduce to a final set of 10 genes. These 10 genes correlated with the survival of the patients, but also with the proliferation index as expressed by *MKI67* gene: MLP, multilayer perceptron; RBF, radial basis function; OS, overall survival; DA, dead/alive; GSEA, gene set enrichment analysis; AUC, area under the curve.

The multilayer perceptron (MLP) analysis was performed as previously described [35,36,37,45,56,57]. The architectures are shown in Figure 1, Figure 2 and Figure 3, and the analysis outline in Figure 4. The MLP procedure produces a predictive model for one or more dependent (target) variables based on the values of the predictor variables. The MLP is a feedforward architecture, the input layer contains the predictors (our gene expression data), the hidden layer contains unobservable nodes or units, and the output layer contains the target variables. The target variables were the overall survival outcome as dead vs. alive, and the gene expression of each prognostic and pathogenic gene as a categorical variable (high vs. low expression). Figure 5, on the top right side, shows the basic neural network architecture. Of note, the basic architecture of the radial basis function (RBF) is like the MLP, but only one hidden layer characterizes it. This research used a simple type of artificial neural network, but solid enough to provide a “basic analysis unit” that conforms a more complex analysis algorithm as shown in Figure 5. A thorough description is shown in our recent publication of artificial analysis of gene expression data of diffuse large b-cell lymphoma (DLBCL) and non-Hodgkin lymphomas [46,58].

#### 2.3.4. Parameters of the Neural Network

A thorough description of the artificial neural network procedure is described in our recent publication [58]. The predictors (covariates) were the 20,862 genes of the array. The covariates were rescaled by default to improve network training. All rescaling was performed based on the training data, even if a testing or holdout sample is defined. The method for rescaling was the standardized (subtract the mean and divide by the standard deviation (x-mean/s)). Other available methods for rescaling were the normalized ((x − min)/(max − min)), adjusted normalized ([2 × (x − min)/(max − min)] − 1), or none. The cases were randomly assigned to the training set, testing set, and holdout according to the relative number of cases, being 70%, 30%, and 0%, respectively. To avoid bias, each individual neural network underwent a random assignation of the samples into the training and testing sets.

The “best” architecture design for the analysis was searched and finally selected [58,59]. The architecture can be selected automatically (with a minimum number of units in the hidden layer of 1 and a maximum of 50) or can be a custom architecture. A custom architecture selection provides control over the hidden and output layers and can be most useful when you know in advance what architecture you want or when you need to tweak the results of the automatic architecture selection.

In a custom architecture, the number of hidden layers could be one or two. The number of units of the hidden layer could be automatically computed or custom. The activation function of the hidden layers was the hyperbolic tangent (γ(c) = tanh(c) = (e^c^ − e^−c^)/(e^c^ + e^−c^)), or sigmoid (γ(c) = 1/(1 + e^−c^)).

The activation function of the output layer was the identity (γ(c) = c), softmax (γ(c_k_) = exp(c_k_)/Σ_j_exp(c_j_)), hyperbolic tangent, or sigmoid. Of note, the activation function chosen for the output layer determined which rescaling methods were available. The rescaling of scale dependent variables was standardized ((x − mean)/s), normalized ((x − min)/(max − min)), adjusted normalized ([2 × (x − min)/(max − min)] − 1), or none.

Several types of training were available: the batch, online, and mini-batch. The optimization algorithm included the scaled conjugate gradient, and gradient descent. The training options were the following: initial lambda (0.0000005); initial sigma (0.00005); interval center (0); and interval offset (±0.5).

The output included the network structure and network performance.

Several parameters displayed the network performance: model summary; classification results; receiver operating characteristic ROC curve; cumulative gains chart; lift chart; predicted by observed chart; and the independent variable importance analysis. ROC analysis displayed a curve for each categorical dependent variable and category and the area under each curve [35,36,37,45,46,56,57]. The predicting variables (predictors) were ranked according to their normalized importance for predicting the target (dependent) variable and for determining the neural network. This analysis performed a sensitivity analysis that is based on the combined training and testing samples or only on the training sample if there is no testing sample [32,33,60].

The predicted value or category and the predicted pseudo-probability for each dependent variable were saved. The synaptic weight estimates were exported to an XML file.

If it was necessary to replicate the results exactly, the same initialization value for the random number generator, data order, and variable order should be used, in addition to using the same procedure settings.

The setup of a radial basis function (RBF) is similar to the MLP. In a RBF, the activation function for hidden layer was normalized or ordinary radial basis function. Figure 1 and Figure 2 show the general architecture for MLP and RBF [32,33,60]. Figure 3 shows the sensitivity analysis [32,33,60]. 

### 2.4. Gene Set Enrichment Analysis (GSEA)

GSEA is a method that determines whether a priori defined set of genes shows statistically concordant differences between two “biological” states (e.g., phenotypes) [48,49]. Three types of files were necessary to run the application: (1) the gene cluster text file (*.gct) with the GSE93291 gene expression dataset; (2) the phenotype data as a categorical class (e.g., dead/alive) file format (*.cls); and (3) the gene set database as a gene matrix file format (*.gmx). The GSEA parameters were the following [37]: number of permutations (1000); collapse to gene symbols; permutation type (phenotype); chip platform (GPL570, HG-U133 Plus 2); enrichment statistic (weighted); metric for ranking genes (signal2noise); gene list sorting mode (real); gene list ordering mode (descending); max size (500); and min size (15) [37].

### 2.5. Summary of the Research Analysis Algorithm

**Figure 6:**
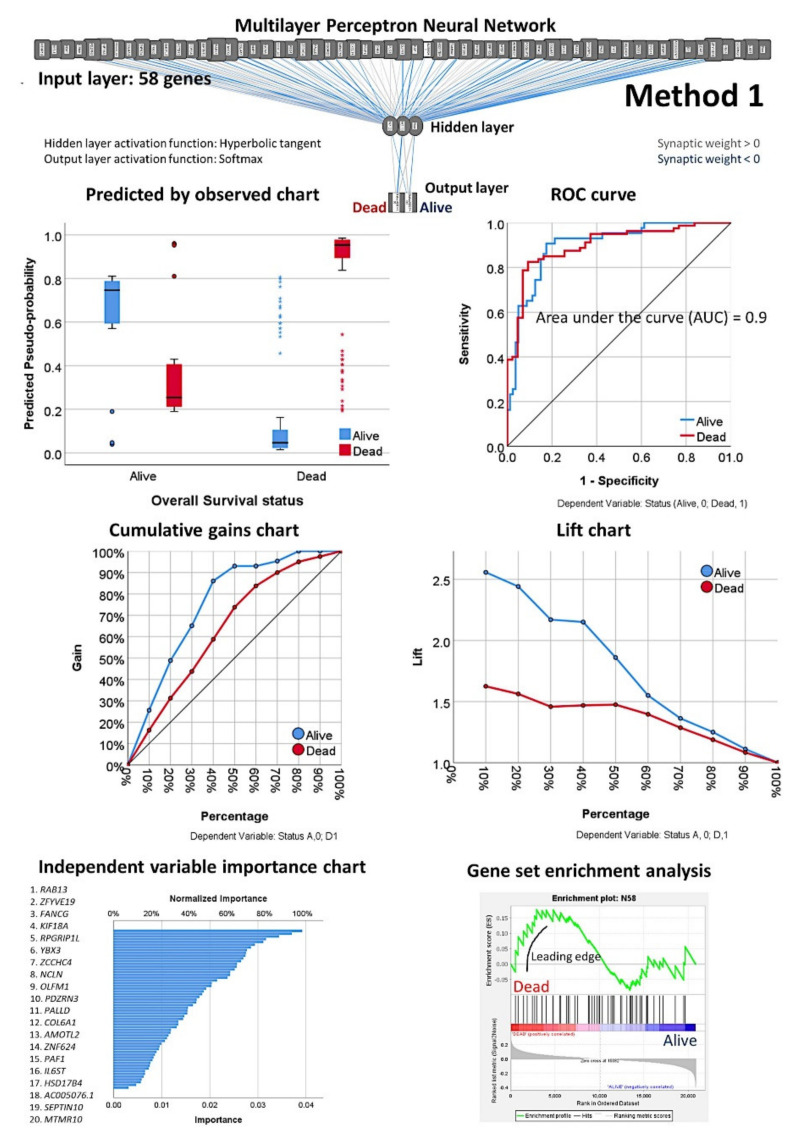
Multilayer perceptron analysis using the selected 58 genes (Method 1 continuation). As shown in Figure 4, the neural networks reduced the initial input of 20,862 genes to 58 predictive genes. Next, the overall survival outcome (dead/alive) was predicted using 58 genes and a neural network. Several parameters display the network performance: model summary; classification results; receiver operating characteristic ROC curve; cumulative gains chart; lift chart; predicted by observed chart; and the independent variable importance analysis. ROC analysis displays a curve for each categorical dependent variable and category and the area under each curve [34,35,36,44,45,55,56]. The genes were ranked according to their normalized importance for predicting the overall survival outcome as a dichotomic variables (dead vs. alive). A GSEA analysis confirmed the association toward a dead outcome. The characteristics of the network were as follows. Case processing: training *n* = 93 (76%); testing *n* = 30 (24%). Units *n* = 58. Rescaling = standardized. Hidden layer: number = 1; units = 2; activation function = hyperbolic tangent. Output layer: dependent variables = 1 (overall survival outcome dead/alive); units = 2, activation function = softmax, error function = cross-entropy. Model summary: training, cross-entropy error = 30.8, 14% of incorrect predictions; testing, cross-entropy error = 14.5, 23% of incorrect predictions. Classification: training, 86% overall correct (93.8% alive, 82% dead); testing, 77% overall correct (82% alive, 74% dead). Area under the curve = 0.9. Top 10 most relevant genes were *RAB13*, *ZFYVE19*, *FANCG*, *KIF18A*, *RPGRIP1L*, *YBX3*, *ZCCHC4*, *NCLN*, *OLFM1*, and *PDZRN3*. A complete description of the multilayer perceptron is present in our recent publication (Carreras J. et al. Artificial Neural Networks Predicted the Overall Survival and Molecular Subtypes of Diffuse Large B-Cell Lymphoma Using a Pan-cancer Immune-Oncology Panel. *Cancers* **2021**, *13*, 6384; https://doi.org/10.3390/cancers13246384) [58].

**Figure 7:**
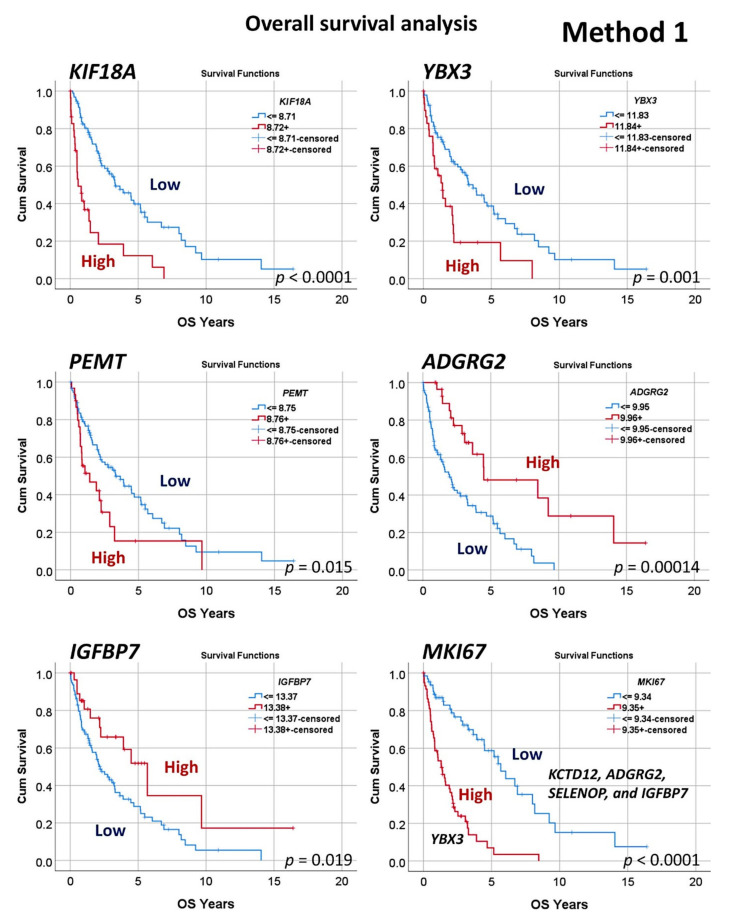
Overall survival analysis (Method 1 continuation). Because of the neural network analysis and dimensional reduction (Figure 4 and Figure 5), a final set of 10 genes with overall survival relationship was highlighted. These genes not only correlated with the clinical outcome but also with the proliferation index, as expressed by *MKI67*. Of note, ki67 is a marker routinely used for prediction in mantle cell lymphoma, and the most relevant marker of the LLMPP MCL35 proliferation assay.

**Figure 8:**
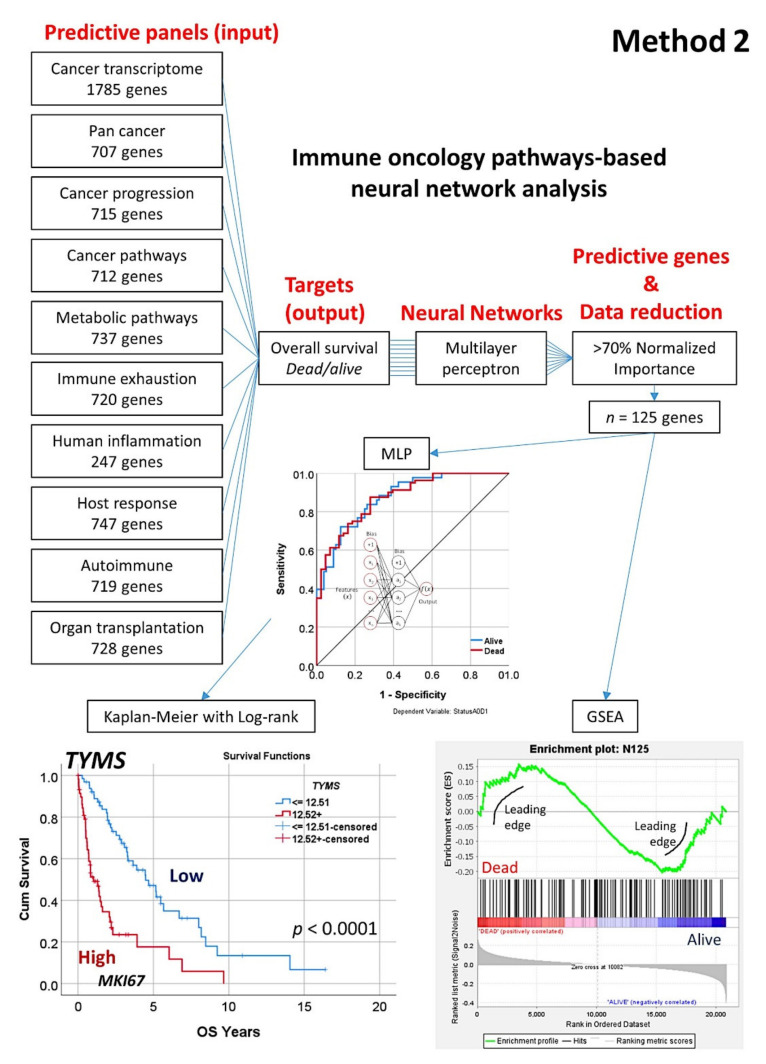
Artificial neural network analysis for predicting of the overall survival of mantle cell lymphoma using several immune oncology panels (Method 2). Overall survival was predicted using 10 immuno-oncology panels. After several multilayer perceptron analyses, a set of 125 genes predicted the overall survival outcome (dead/alive) with high accuracy. Among the most relevant genes, *TYMS* was highlighted. GSEA analysis had a sinusoidal-like, with some genes enriched toward dead or alive survival outcomes.

The algorithms for the analysis of the gene expression data of MCL are shown in Figure 5, Figure 6, Figure 7 and Figure 8.

#### 2.5.1. Algorithm Based on the Input of 20,862 Genes (Method 1)

First, all the genes of the array were used as predictors (input layer) for the target variables (output layer) of overall survival (dead/alive) and for the 28 genes with prognostic value in MCL (high/low expression) using an artificial neural network. The neural network included both a multilayer perceptron and a radial basis function analysis for each target variable (Figure 5). In the output of each individual neural network, all the genes of the array were ranked according to their normalized importance for predicting the target variable. Then, the genes with a normalized importance above 70% were selected. In addition, the normalized importance of all the neural networks were averaged, the genes ranked according to the averaged normalized importance for prediction, and the top 1% genes were selected. As a result, the initial set of 20,862 genes was reduced to a smaller number (*n* = 1394).

Next, an MLP was performed using the 1394 genes as predictors (input layer) of the overall survival outcome (dead/alive, output layer); this analysis was repeated 20 times, and the top 4 MLPs with higher area under the curves were selected. The normalized importance of each 1394 were averaged between the four results and ranked from higher to lower values. Then, using multiple MLP analysis, the minimum number of genes (starting from the one with higher normalized importance) that provided the highest area under the curve was found (*n* = 58) (Figure 6).

**Figure 9:**
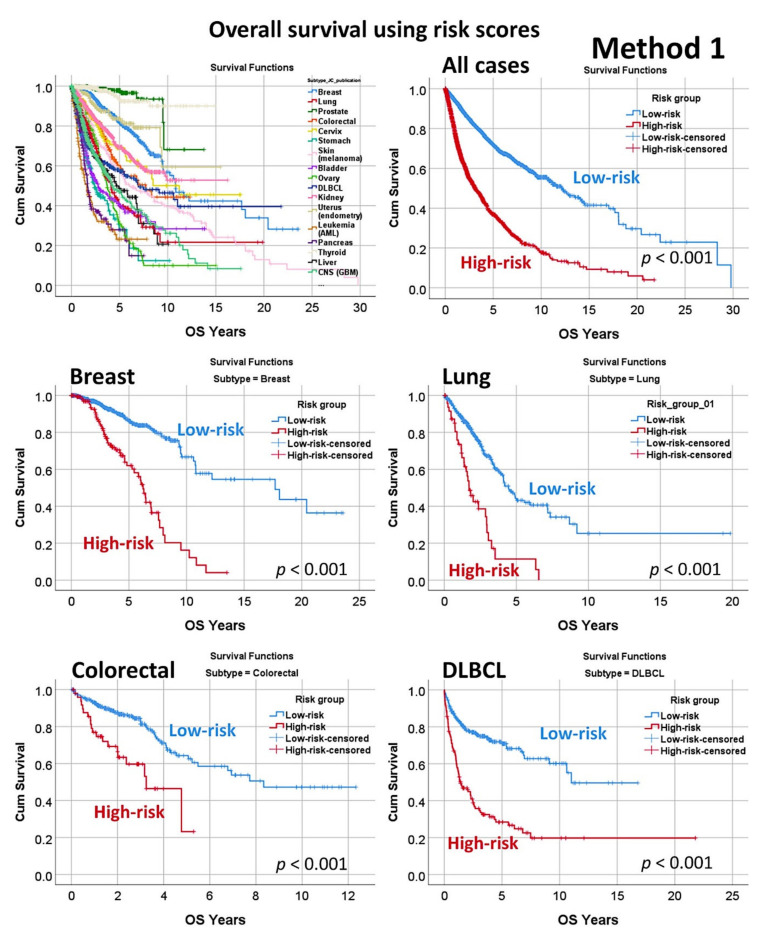
Overall survival in a pan-cancer series. The multilayer perceptron using the 20,862 genes identified a final set of 19 genes with prognostic value in mantle cell lymphoma. As a start point of the gene expression of the set of 19 genes and using a risk-score formula [36,46], we confirmed that these genes also contributed to the overall survival of diffuse large B-cell lymphoma (DLBCL). Additionally, these genes could also predict the overall survival of a pan-cancer series of 7289 cases from The Cancer Genome Atlas (TCGA) program that included the most frequent human cancers. Of note, the weight and direction of the overall survival association was different in each subtype of neoplasia. Risk scores were calculated by multiplying the beta values of the multivariate Cox regression analysis for overall survival of each gene with the values of the corresponding gene expressions, as previously described [58].

**Figure 10:**
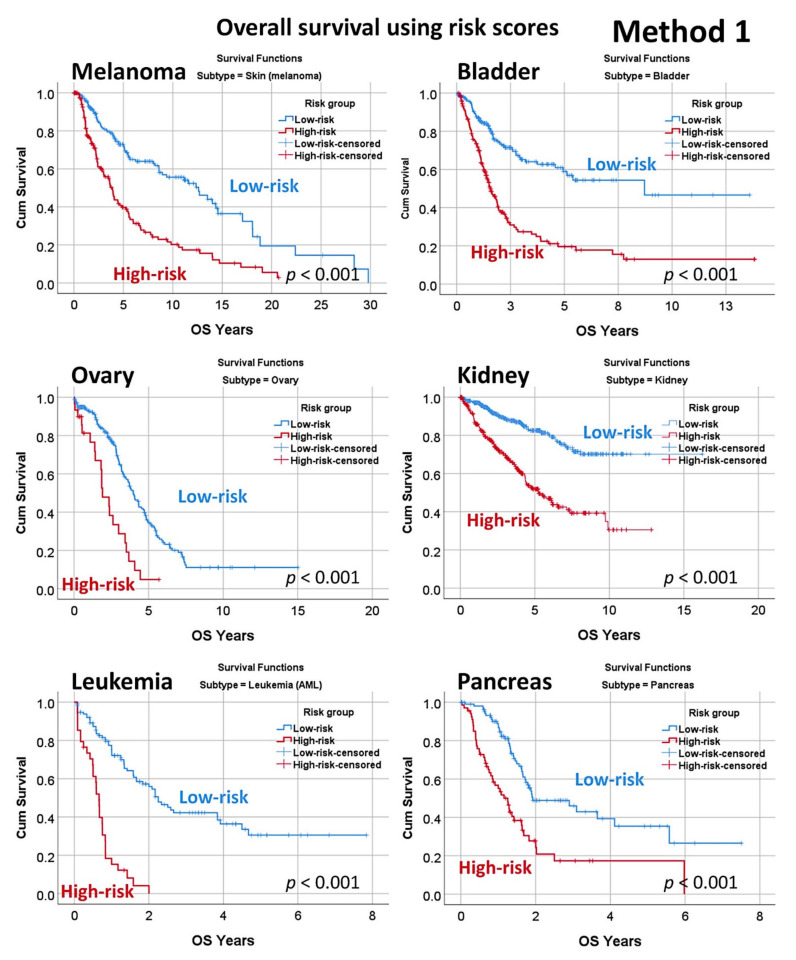
Overall survival in a pan cancer series.

**Figure 11:**
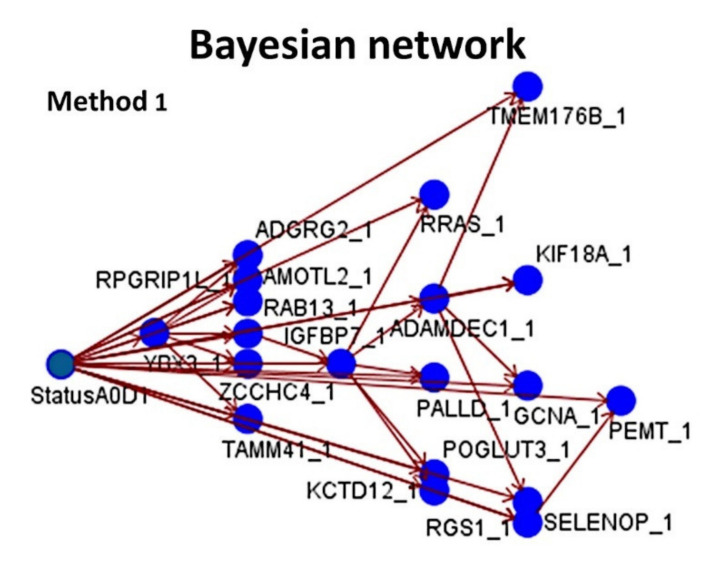
Bayesian network. A Bayesian network successfully modeled the overall survival outcome (dead/alive) using the 19 genes, previously identified in the neural network analysis (Figure 5, Method 1). The Bayesian network enables you to build a probability model by combining observed and recorded evidence with “common-sense” real-world knowledge to establish the likelihood of occurrences by using seemingly unlinked attributes. The node focuses on Tree Augmented Naïve Bayes (TAN) and Markov Blanket networks that are primarily used for classification. This graphical model shows the variables (nodes) and the probabilistic, or conditional, independencies between them. The links of the network (arcs) may represent causal relationships, but the links do not necessary represent direct cause and effect. This Bayesian network is used to calculate the probability of a patient of being alive or dead, given the gene expression of 19 genes, if the probabilistic independencies between the gene expression and the overall survival outcome as displayed on the graph hold true. Bayesian networks are very robust in case of missing data.

**Figure 12:**
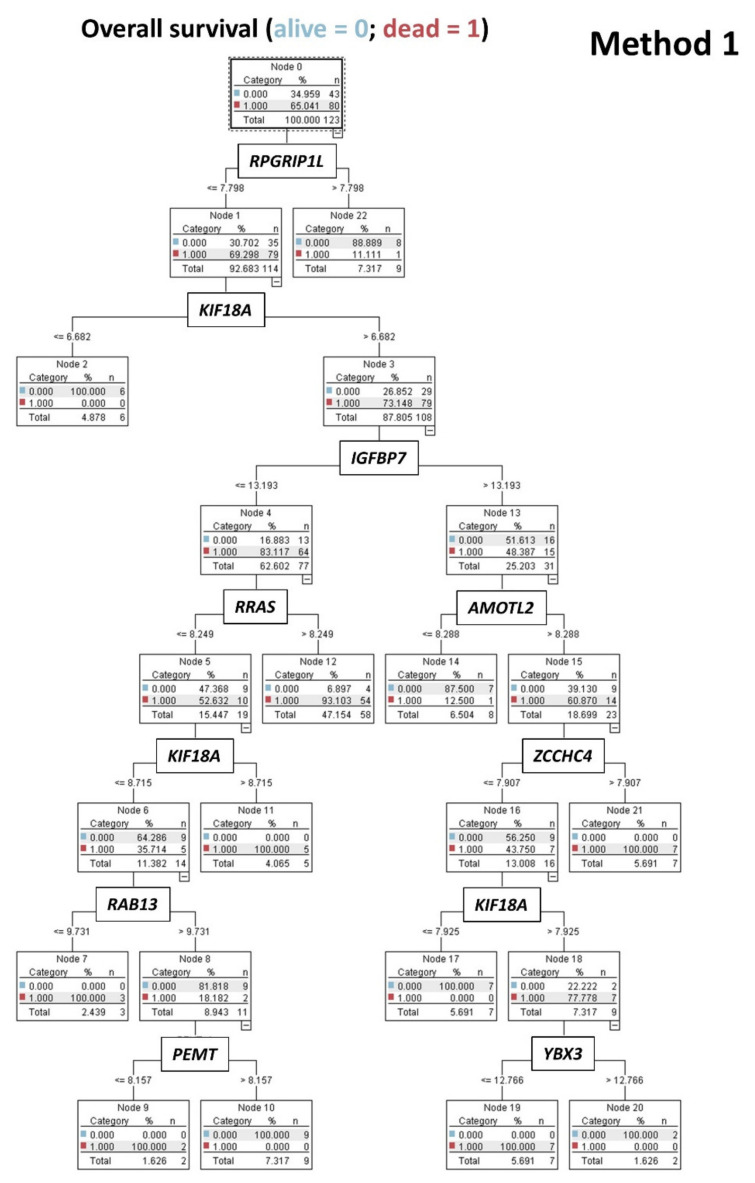
C5.0 decision tree model. A decision tree successfully modeled the overall survival outcome (dead/alive) using the 19 genes, previously identified in the neural network analysis (Figure 5, Method 1). This model uses the C5.0 algorithm to build either a decision tree or a rule set. A C5.0 model works by splitting the sample based on the field that provides the maximum information gain. Each subsample defined by the first split is then split again, usually based on a different field, and the process repeats until the subsamples cannot be split any further. Finally, the lowest-level splits are reexamined, and those that do not contribute significantly to the value are removed. In this model, the target field (variable) must be categorical (i.e., nominal or ordinal, such as de overall survival outcome as dead vs. alive). The input fields (predictors) can be of any type (in our analysis, the 19 genes were entered as quantitative gene expression). The C5.0 models are quite robust in the presence of problems such as missing data and large numbers of input fields. The C5.0 tree shows how using only the gene expression of 9 genes, the overall survival outcome as dead or alive can be predicted with high accuracy.

**Figure 13:**
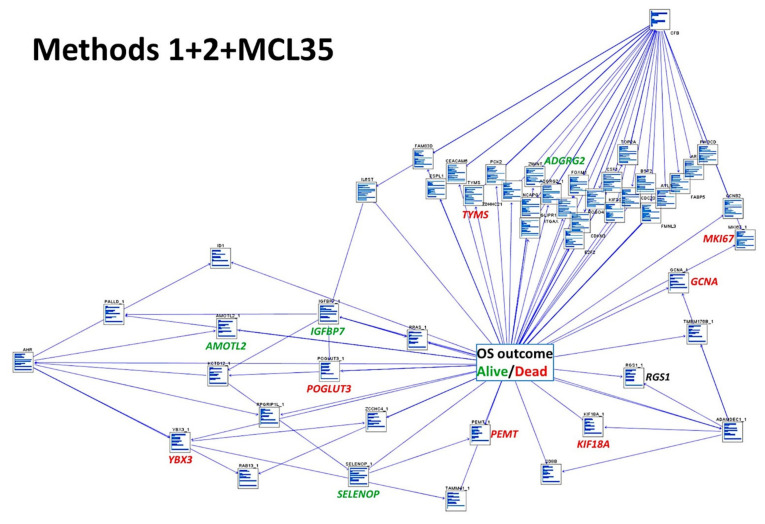
Addition of the MCL35 proliferation signature in a Bayesian network. A Bayesian network modeling was performed using the highlighted genes of both Methods 1 (19 genes) and Methods 2 (15) with the previously identified prognostic genes of MCL of the LLMPP, the MCL35 signature. Some of the most relevant genes are highlighted, in red for the bad, green for the good prognostic genes, and their interrelationships (arrows).

**Figure 14:**
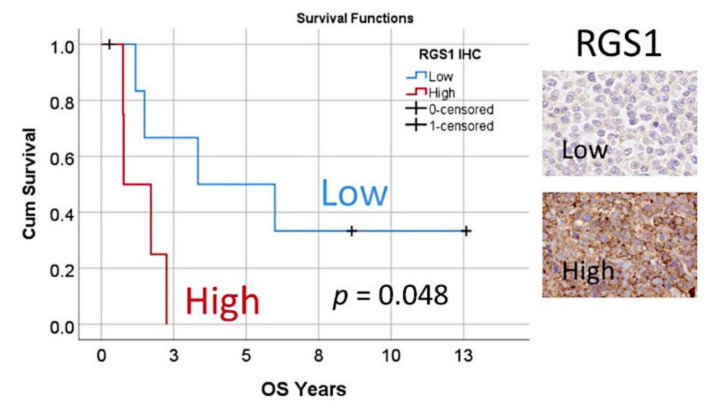
Overall survival according to the immunohistochemical expression of RGS1.

Finally, a Cox regression for overall survival (backward conditional) reduced the list to 19 genes. From these 19 genes, additional analyses included Kaplan–Meier with log-rank test for overall survival using cutoffs (Figure 7), analysis of other types of cancer (“pan-cancer analysis”) (Figure 9 and Figure 10), other machine learning (Figure 11, Figure 12 and Figure 13), and immunohistochemistry for RGS1 (Figure 14).

#### 2.5.2. Algorithm Based on the Input of 10 Immune Oncology Panels (Method 2)

In comparison to the first algorithm in which the whole genes of the array were used (*n* = 20,862), this second algorithm used 9 different immune oncology panels as input data (7817 genes in total) (Figure 8). Nine individual MLP analysis for the prediction of overall survival outcome (dead/alive) were performed, and the genes with a normalized importance above 70% in each panel were pooled (*n* = 125). A GSEA analysis confirmed the association of these genes towards the dead or alive overall survival outcome (phenotype). Next, an additional MLP analysis confirmed the prediction of the overall survival outcome and ranked the 125 genes according to their normalized importance. The top genes were later tested for conventional overall survival analysis.

### 2.6. Conventional Statistical Analyses

Traditional statistics calculated the overall survival analyses. Overall survival was calculated from time of diagnosis to the last follow-up time, and recorded as alive or dead (event), following the criteria of Cheson B. D. [61,62]. Comparison between groups was performed using Kaplan–Meier analysis and the log-rank test. The Breslow and Tarone–Ware tests were also used. The Cox regression (with the method enter or backward conditional) was used to calculate the hazard-risks and the 95% confidence intervals. A *p* value less than 0.05 was considered statistically significant.

In case of a neural network analysis, poor prognosis/survival corresponds to the cases whose overall survival event was dead. In case of an overall survival analysis using the Kaplan–Meier test, poor prognosis corresponds to the group with lower cumulative survival proportion in the plot.

### 2.7. Immunohistochemistry

The immunohistochemistry was performed using an automated piece of equipment, Leica BOND-MAX stainer, following the manufacturer’s instructions and as previously described [53,59,63,64,65]. The RGS1 primary antibody (rabbit polyclonal) was purchased from Thermofisher [63]. The slides were digitalized using a Hamamatsu NanoZoomer S360, scanned, and visualized using the NDP.veiw2 software. 

## 3. Results

### 3.1. Highlights

**Figure A1:**
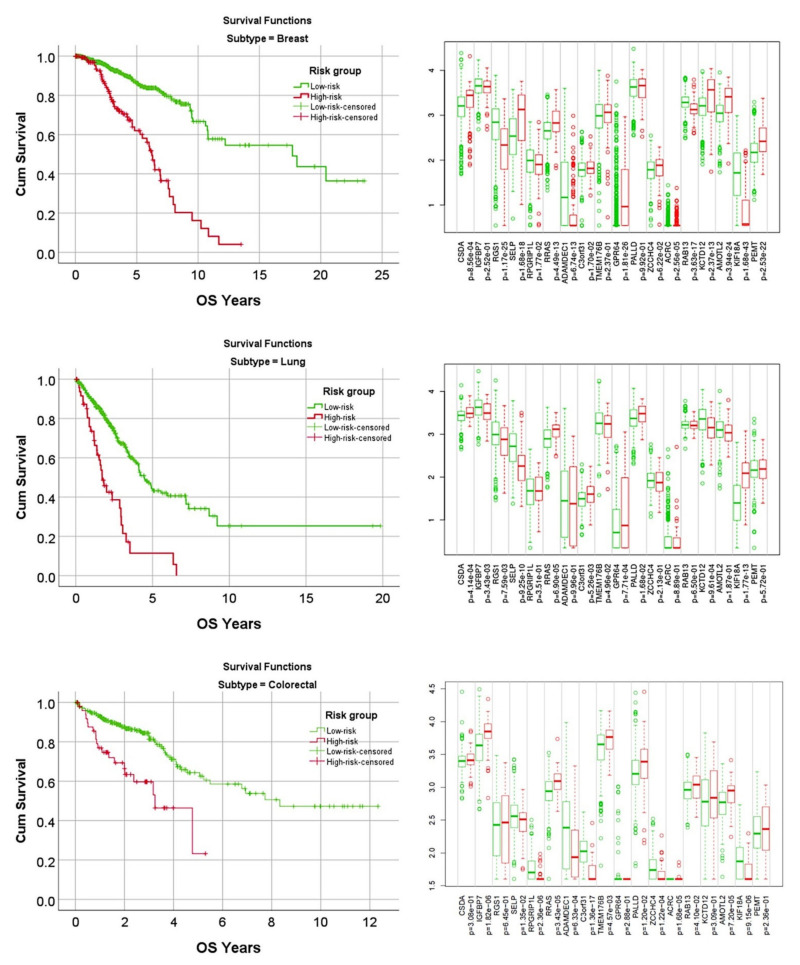
Differential gene expression of the set of 19 genes per cancer subtype. Based on a risk-score formula and the gene expression of 19 genes, the overall survival for each risk-group could be calculated. The contribution in the prognosis for each gene is shown on the right. This Figure is complementary to Figure 9.

- Using 20,862 genes as a start point (input layers) (Method 1), several neural network analyses correlated with the overall survival outcome and with known pathogenic genes of MCL (output layers), and a final set of 19 genes with predictive value was highlighted (Figure 5);
- This type of analysis was repeated focusing on 10 immune, cancer, and immuno-oncology panels (Method 2), and 15 genes were highlighted (Figure 8);
- Other machine learning techniques were used to predict the overall survival (Figure 11 and Figure 12);
- The highlighted genes also predicted the overall survival of a pan-cancer series (Figure 9, Figure 10 and Figure A1);
- The combination of both Methods 1 (19 genes) and 2 (15 genes) with the LLMPP MCL35 assay (17) genes and analysis using several machine learning and neural networks techniques predicted the overall survival outcome (dead vs. alive) with high accuracy.

### 3.2. Prediction of Overall Survival Based on the 20,862 Genes of the Array (Method 1)

Dimensionality reduction refers to techniques for reducing the number of input variables in training data. Fewer input dimensions often mean correspondingly fewer parameters or a simpler architecture in the machine learning model, referred to as degrees of freedom [66]. The input layer of 20,862 predicted the overall survival of mantle cell lymphoma (MCL), using an analysis algorithm (Figure 5). The output variables (targets) were the overall survival outcome as a dichotomous variable (dead/alive), and the 28 genes (high/low expression) with prognostic relevance for the overall survival were confirmed in the same series (Table 2). Table A1 and Table A2 show the complete details of the artificial neural networks. The multilayer perceptron (MLP) technique had better performance than the radial basis function (RBF): comparing area under the curve, percentage of incorrect predictions (testing set), and overall percentage of correct classification (testing set), for MLP vs. RBF, the results were 0.85 ± 0.05 vs. 0.77 ± 0.09 (*p* = 0.000053), 15.3% ± 5.9 vs. 26.5% ± 10.2 (*p* = 0.000005), and 84.7% ± 5.9 vs. 73.5% ± 10.2 (*p* = 0.000005), respectively. *CCND1* was the best predicted gene; in the MLP analysis *CCND1* had a percentage of incorrect predictions in the testing set of 2.8%, the lowest value among all genes (Table A1).

From the initial 20,862 genes, the list was reduced to 1394 genes, and additional multilayer perceptron analyses led to a set of 58 genes (Figure 6). The network performance of the MLP with the input of 58 genes was “good”, with an area under the curve (AUC) of 0.9. The genes were ranked based on their normalized importance for prediction, and GSEA confirmed that most of these genes were associated with the death survival outcome (Figure 6); the most relevant were *KIF18A*, *FANCG*, *GCNA*, *YBX3*, *ZCCHC4*, and *DMTF1.*

Based on the 58 genes, a subsequent multivariate Cox regression analysis, backward conditional, highlighted a set of 19 genes (Table A3), and a final set of 10 genes was found after using a cut-off and a Kaplan–Meier analysis for overall survival (Table 2). *KIF18A*, *YBX3*, *PEMT*, *GCNA*, and *POGLUT3* were associated with an unfavorable overall survival, and *SELENOP*, *AMOTL2*, *IGFBP7*, *KCTD12*, and *ADGRG2* to a favorable survival (Figure 6). Finally, the 10 genes were correlated with the cell proliferation marker of *MKI67*, which is one of the most relevant genes in the pathogenesis of MCL (Table 3). The cases with low *MKI67* were associated with high *KCTD12*, *ADGRG2*, *SELENOP*, and *IGFBP7*. However, high *MKI67* associated with high *YBX3*. Table A4 shows a multivariate analysis for overall survival between *MIK67* and the 10 genes using a Cox regression.

Therefore, the dimensionality/data reduction of the Methods 1 went from 20,862 initial genes, to 1394, 58, 19, and the final 10 most relevant prognostic genes for overall survival of MCL patients.

### 3.3. Prediction of Overall Survival Based on the Immuno-Oncology Panels (Method 2)

The prediction of the overall survival outcome was performed using another strategy, based on nine different immune oncology pathways, multilayer perceptron neural networks, GSEA, and Kapan–Meier analyses (Figure 8).

The characteristics and performance parameters of the neural networks are shown in Table A5. The most predictive panels (pathways) were the autoimmune (AUC = 0.98), the pan cancer human IO360 (AUC = 0.94), human inflammation (AUC = 0.89), pan cancer (AUC = 0.89), and metabolic (AUC = 0.87). Interestingly, some pathways had a more predictive power toward the dead than the alive outcome.

After selecting the genes with a normalized importance above 70% and merging, a final set of 125 was identified. A GSEA on these 125 genes had a sinusoidal-like pattern, with some genes associated toward poor (dead) and others to favorable (alive) overall survival. The genes were ranked according to their normalized importance for prediction using a multilayer perceptron analysis, and the top 15 genes were *CD8B*, *CEACAM6*, *FABP5*, *CFB*, *IL6ST*, *AHR*, *BST2*, *ROBO4*, *AR*, *ID1*, *PIK3CD*, *ITGAX*, *TYMS*, *CSF1*, and *PCK2* (normalized importance >0.68). Among them, *TYMS* was highlighted, and this gene by itself managed to predict the overall survival of the patients (Hazard risk (HR) = 3.2, 95% CI 2.0–5.0, *p* = 8.9 × 10^−7^). Of note, high *TYMS* also correlated with high *MIK67* expression (Fisher’s exact test, *p* = 0.001).

In a multivariate Cox regression survival analysis including these top 15 genes as quantitative variables, backward conditional method, in the last step (11) the significant genes were *TYMS* (*p* < 0.001, HR = 2.6), *AR* (*p* = 0.012, HR = 1.5), and *CSF1* (*p* = 0.049, HR = 0.6).

### 3.4. Prediction of Overall Survival of a Pan-Cancer Series

The predictive value of the set of 19 genes, derived from neural network analysis and dimensional reduction of the initial 20,862 genes (Figure 5, Method 1), was tested for the prediction of a pan cancer series of 7289 cases from The Cancer Genome Atlas (TCGA) database and GSE10846 dataset for diffuse large B-cell lymphoma (DLBCL). Using a risk-score formula [36,46], a different overall survival of the patients was found, confirming the pathological role of these genes in cancer (Figure 9 and Figure 10, Table A6, Figure A1). In overall high-risk versus low-risk cases, Cox regression hazard risk = 3.3 (95% CI 2.9–3.6), *p* < 0.0001.

### 3.5. Prediction of Overall Survival Outcome Using other Machine Learning Techniques

The predictive value of the set of 19 genes (Method 1) as quantitative variables for the overall survival outcome was modeled using other machine-learning techniques, including logistic regression, Bayesian network, discriminant analysis, KNN algorithm, LSVM, tree-AS, C5, CHAID, Quest, random, and C&R trees. Among them, the highest overall accuracy for prediction was achieved by the C5 tree (95%, 9 genes used), and Bayesian network (85%, 19 genes, Figure 11 and Figure 12).

### 3.6. Combination of Method 1, Method 2, and the LLMPP MCL35 Prognostic Gene Signature

A machine learning and neural network modeling was performed using the highlighted genes of both Methods 1 (19 genes) and Methods 2 (15) with the previously identified prognostic genes of MCL of the LLMPP, the MCL35 signature [50,67,68,69]. All the available artificial intelligence methods were tested, and high overall accuracy for predicting was found for logistic regression (100%), Bayesian network (92%), discriminant analysis (86%), CHAID (85%), C&R tree (85%), and SVM (81%) (Table 4, Figure 13).

### 3.7. Immunohistochemical Analysis of RGS1

RGS1 was identified as an MCL prognostic gene. It was present within the set of 19 in the last step of the first analysis algorithm (Figure 5) and the Cox regression (backward conditional). The prognostic association was tested by immunohistochemistry in a series of 11 cases of MCL from Tokai University. Among the different gene candidates, *RGS1* was selected because a reliable primary antibody for immunohistochemistry was available, and we previously showed that high RGS1 protein expression correlated with poor prognosis in diffuse large b-cell lymphoma [63]. The clinicopathological characteristics of this series was the following: age (median, 72 years; range 41–82); male (9/11, 82%); lymph node and tonsil biopsy (10/11, 91%); CD3-negative (100%); CD5-positive (10/11, 91%); CD20, CD10, Cyclin D1 (*CCND1*) and BCL2-positive (100%); BCL6-positive (3/11, 27%); MUM-1(*IRF4*)-positive (9/10, 90%); proliferation index (Ki67, 10–50%).

The RGS1 protein expression was evaluated as low and high, and correlated with the overall survival of the patients (*p* = 0.048) (Figure 10). Nevertheless, no correlation was found between RGS1 and the other clinicopathological characteristics.

## 4. Discussion

Mantle cell lymphoma is a hematological neoplasia that belongs to the group of non-Hodgkin lymphomas (NHL) and it is derived from mature B-lymphocytes [16].

The postulated cell of origin in most of the cases is a naïve pregerminal center B-cell of the mantle zone [1,9,16,17,46], because of the absence of somatic mutations in the variable region of the heavy chain of immunoglobulin genes (*IgVH*). *IgVH* somatic mutational status is a marker of the transition of a B-lymphocyte through a follicular germinal center [70]. However, in 20–30% of the cases somatic hypermutation is found, which suggests a postgerminal origin (marginal zone) [71], and these cases are associated with a better prognosis [72]. Because of the aggressive clinical behavior of mantle cell lymphoma, it is critical to find prognostic makers that will allow identifying the patients who should receive more aggressive therapy.

Mantle cell lymphoma is characterized by increased cell division and replication, decreased response to DNA damage, and enhanced cell survival (impaired apoptosis) [16]. Some of these pathways and genes correlate with prognosis. For instance, *TP53* and *NOTCH1* mutations, overexpression of *SOX11*, and high proliferation index (Ki67 staining) associate with a poor prognosis.

This research identified new prognostic markers using gene expression data. Dimensionality reduction refers to techniques for reducing the number of input variables in training data. Fewer input dimensions often mean correspondingly fewer parameters or a simpler architecture in the machine learning model, referred to as degrees of freedom [66]. A neural network analysis correlated the 20,862 genes of the array with the overall survival outcome (dead/alive), and ranked the genes according to their normalized importance for prediction. Additionally, the analysis was enriched with the inclusion of 28 prognostic genes, which were identified from the literature and later confirmed to have prognostic relevance in this series (Table 1). Therefore, the input data of the neural network were solid and resulted in the identification of potentially relevant new prognostic markers. Additionally, the second type of neural network analysis was performed using several immune oncology pathways, which provided a more supervised training and analysis. The fact that we found a correlation of some of the highlighted genes with the expression of *MKI67*, a marker of proliferation known to be critical in mantle cell lymphoma pathogenesis, suggests that the identified new markers are also potentially relevant.

The highlighted genes influence apoptosis, angiogenesis, cell proliferation, and metabolic processes. They contribute to hematological neoplasia or cancer (Table 5). Therefore, it is expected that these genes also affect the progression of the pan cancer series.

It is important to point out that one could also use background information (e.g., patient age, sex, comorbidities, etc.) into the artificial neural network analyses. Incorporating such information would have a large impact on the results. In this research, the target was the prediction of the overall survival of patients based on the gene expression data as proof of concept. In future analyses, background information will be incorporated in MCL analysis, in a similar way as we have recently done in diffuse large b-cell lymphoma (DLBCL) [35].

In addition to neural networks, other machine learning techniques were tested, and the C5 tree and Bayesian networks had the best accuracy for predicting the overall survival outcome. Of note, the type of analyses used do not necessarily represent direct cause and effect, but the probabilistic or conditional independencies between the markers.

The recent advances in machine learning have led to many artificial intelligence (AI) applications, which will produce autonomous systems. However, the effectiveness of these systems is limited by the machine’s current inability to explain their decision and actions to human users [87]. Therefore, explainable AI (XAI) will be essential to understand, trust, and effectively managed AI machine partners [87]. In this research, the artificial neural networks highlighted the most relevant genes according to their normalized importance for predicting the overall survival of the patients. To make the results more explainable, we performed serval additional machine learning techniques and conventional statistics to understand the results. For future work, the explanation of algorithms will be developed. Of note, in medicine, AI technologies can be clinically validated even when their function cannot be understood by their operators [88].

Future research directions will be the validation of the methodology and highlighted genes in other series of mantle cell lymphoma and non-Hodgkin lymphomas.

## 5. Conclusions

This research combined artificial neural networks, machine learning, and conventional statistics to model the overall survival of mantle cell lymphoma and highlight pathogenic genes. Artificial intelligence is a promising field in the understanding of hematological neoplasia, and other types of cancer.

## Figures and Tables

**Table 1:** Prognostic and pathogenic genes of mantle cell lymphoma.

Genes (*n* = 86)
*ADAMDEC1*, *ADGRG2*, *AKT1*, *AKT3*, *AMOTL2*, *ARID2*, *ATM*, *BCL2*, *BCL2L11*, *BCL6*, *BCOR*, *BIRC3*, *BMI1*, *BORCS8_MEF2B*, *BTK*, *CARD11*, *CASP8*, *CCND1*, *CCND2*, *CCND3*, *CD5*, *CD79A*, *CDK4*, *CDKN1B*, *CDKN2A*, *CDKN2C*, *CFLAR*, *CHEK1*, *CHEK2*, *CUL4A*, *CXCL12*, *CXCR4*, *DAZAP1*, *GCNA*, *HNRNPH1*, *IGFBP7*, *ING1*, *KCTD12*, *KIF18A*, *KMT2C*, *KMT2D*, *LYN*, *MDM2*, *MIR17HG*, *MKI67*, *MTOR*, *MYC*, *MYCN*, *NFKB1*, *NFKBIE*, *NOTCH1*, *NOTCH2*, *NSD2*, *PALLD*, *PAX5*, *PDGFA*, *PEMT*, *PIK3CA*, *PIK3CD*, *POGLUT3*, *PTEN*, *PTK2*, *RAB13*, *RB1*, *RGS1*, *RPGRIP1L*, *RRAS*, *SAMHD1*, *SELENOP*, *SMARCA2*, *SMARCA4*, *SMARCB1*, *SOX11*, *SYK*, *SYNE1*, *TAMM41*, *TERT*, *TET2*, *TMEM176B*, *TNFAIP3*, *TP53*, *TRAF2*, *UBR5*, *XIAP*, *YBX3*, and *ZCCHC4*

Eighty-six genes with predictive and pathogenic role in MCL were selected from the literature. These genes were later tested for overall survival in the GSE93291 series. Only significant ones were chosen for the neural network analysis.

**Table 2:** Pathogenic genes of mantle cell lymphoma (GSE93291 series) (Method 1).

Gene	Keyword	Function	Correlation with the Overall Survival of MCL		
			*beta*	*p*	HR
*BCL2L11*	Apoptosis	B-cell apoptotic process	1.0	<0.01	2.7
*BMI1*	Regulation of gene expression	Component of the Polycomb group (PcG) multiprotein PRC1-like complex, negative regulation of gene expression, epigenetic	−0.5	0.042	0.6
*BORCS8_MEF2B*	Lysosomes	BORC complex, role in lysosomes movement and localization at the cell periphery	−1.0	<0.01	0.4
*CCND1*	Cell cycle	Positive regulation of G1/S transition of the mitotic cell cycle	1.1	<0.01	3.1
*CCND2*	Cell cycle, apoptosis	Positive regulation of G1/S transition of the mitotic cell cycle, negative regulation of apoptosis	−0.7	0.018	0.5
*CDK4*	Cell cycle, apoptosis	Negative regulation of G1/S transition of the mitotic cell cycle, positive regulation of apoptotic process	1.4	<0.01	4.0
*CDKN2A*	Cell cycle, NF-kB, apoptosis	Negative regulation of G1/S transition of the mitotic cell cycle, negative regulation of NF-kB, positive regulation of apoptotic process	1.0	<0.01	2.7
*CDKN2C*	Cell cycle	Negative regulation of G1/S transition of the mitotic cell cycle	1.0	<0.01	2.8
*CHEK1*	Cell cycle, DNA repair, apoptosis	Positive regulation of cell cycle, DNA damage checkpoint and repair, apoptosis	1.1	<0.01	3.0
*CHEK2*	Cell cycle, DNA repair, apoptosis	Positive regulation of cell cycle, DNA damage checkpoint and repair, apoptosis	0.8	<0.01	2.1
*CXCL12*	Chemotaxis, apoptosis	Cell chemotaxis, defense response, negative regulation of apoptotic process, DNA damage	−0.6	0.014	0.5
*DAZAP1*	Cell differentiation and proliferation	Cell differentiation, cell proliferation, positive regulation of mRNA splicing	0.8	0.016	2.3
*ING1*	Cell cycle	Negative regulation of cell growth, cooperates with TP53	−1.1	<0.01	0.3
*MKI67*	Cell proliferation	rRNA transcription	1.5	<0.01	4.4
*MYC*	Cell proliferation	Transcription factor that binds DNA and activates transcription of growth-related genes (positive regulation of gene expression), negative regulation of apoptotic process	0.9	<0.01	2.5
*MYCN*	Gene expression	Regulation of gene expression, DNA-binding	−0.5	0.052	0.6
*NOTCH1*	Multiple negative regulations	Affects the implementation of differentiation, proliferation, angiogenesis, and apoptotic programs. Multiple negative regulations	−0.8	<0.01	0.5
*NOTCH2*	Multiple regulations	Affects the implementation of differentiation, proliferation and apoptotic programs	0.6	0.020	1.8
*NSD2*	B-cell development	Histone methyltransferase, B-cell development (B1), and B2 activation, humoral immune response, isotype class switch recombination, germinal center formation	1.0	<0.01	2.7
*PAX5*	B-cell development	The commitment of lymphoid progenitors to B-lymphocyte lineage, promotes development of the mature B-cell stage.	−0.7	0.010	0.5
*PIK3CA*	ERBB2 signaling, apoptosis	Cell migration, ERBB2 signaling pathway, negative regulation of apoptosis,	0.5	0.042	1.7
*PIK3CD*	B-cell development and function	Mediates immune responses. Contributes to B-cell development, proliferation, migration, and function. Required for B-cell receptor (BCR) signaling	0.5	0.025	1.7
*PTEN*	Cell cycle, tumor suppressor gene	Negative regulation of G1/S transition of the mitotic cell cycle	−0.8	0.012	0.5
*PTK2*	Multiple regulations	Regulation of cell migration, adhesion, cell cycle progression, cell proliferation, apoptosis, MAPK/ERK1 pathway, MDM2 and TP53 recruitment	0.5	0.035	1.7
*RB1*	Cell cycle, tumor suppressor gene	Tumor suppressor that is a key regulator of the G1/S transition of the cell cycle	−0.5	0.043	0.6
*SYNE1*	Cytoskeleton	Cytoskeleton-nuclear membrane anchor activity, maintaining of subcellular spatial organization	−0.6	<0.01	0.5
*TERT*	Telomerase, multiple functions	Telomerase, negative regulation apoptosis, positive regulation G1/S transition of the mitotic cell cycle, negative regulation of gene expression	0.7	<0.01	2.0
*XIAP*	Multiple functions, regulation of caspases and apoptosis	Multi-functional protein that regulates not only caspases and apoptosis, but also modulates inflammatory signaling and immunity, copper homeostasis, mitogenic kinase signaling, cell proliferation, as well as cell invasion and metastasis	−0.8	<0.01	0.5

From an initial set of 86 genes with known pathogenic role in MCL, a final set of 28 genes were selected because their predictive value for overall survival using a Kaplan–Meier and log-rank test in the GSE93291: *P*, *p* value; HR, hazard risk. The gene information is based on UniProt [54], and Genecards [55].

**Table 3:** Kaplan–Meier analysis for prediction of overall survival outcome (Method 1).

m	Gene	Cut-Off	Log-Rank *p* Value	Breslow *p* Value	Hazard Risk	Correlation with High *MKI67*, Odds Ratio (OR)	OR *p* Value
1	*KIF18A*	8.71	<0.001	<0.001	3.5 (2.1–5.8)	1.3 (0.6–3.0)	0.499
2	*YBX3*	11.83	0.001	0.002	2.3 (1.4–3.8)	2.3 (0.9–5.3)	0.056
3	*PEMT*	8.75	0.015	0.016	1.9 (1.1–3.1)	1.1 (0.5–2.5)	0.798
4	*GCNA*	7.66	0.037	0.137	1.8 (1.0–3.3)	2.1 (0.9–4.9)	0.077
5	*POGLUT3*	8.81	0.034	0.014	1.6 (1.0–2.5)	0.9 (0.4–1.7)	0.649
6	*SELENOP*	12.81	0.028	0.048	0.6 (0.4–0.9)	0.2 (0.1–0.5)	0.001
7	*AMOTL2*	8.99	0.039	0.029	0.5 (0.3–0.9)	0.5 (0.2–1.1)	0.068
8	*IGFBP7*	13.37	0.019	0.042	0.5 (0.3–0.9)	0.2 (0.1–0.4)	<0.001
9	*KCTD12*	12.02	0.022	0.042	0.5 (0.3–0.9)	0.2 (0.1–0.5)	0.01
10	*ADGRG2*	9.95	<0.001	<0.001	0.3 (0.2–0.6)	0.2 (0.1–0.5)	0.001

This analysis is a univariate.

**Table 4:** Machine learning and neural network analysis of the combined Methods 1 and 2 with the MCL35 signature.

Model	Overall Accuracy for Predicting the Overall Survival	No. of Genes Used in the Final Model	Gene Names
Logistic regression	100	50	All the 50
Bayesian network	92	50	All the 50
Discriminant	86	50	All the 50
CHAID	85	6	*E2F2*, *GCNA*, *FMNL3*, *POGLUT3*, *SELENOP*, and *ZDHHC21*
C&R tree	85	21	*ADGRG2*, *CDC20*, *CEACAM6*, *ESPL1*, *FABP5*, *FAM83D*, *FMNL3*, *GCNA*, *GLIPR1*, *ID1*, *ITGAX*, *KIF2C*, *MKI67*, *RGS1*, *ROBO4*, *RPGRIP1L*, *RRAS*, *SELENOP*, *TAMM41*, *ZDHHC21*, and *ZWINT*
SVM	81	50	All the 50
KNN algorithm	78	50	All the 50
Neural network	76	50	All the 50
C5	76	3	*ESPL1*, *RPGRIP1L*, and *ZWINT*
Quest	65	50	All the 50

In this analysis, several methods were tested, including C5, logistic regression, Bayesian network, discriminant analysis, KNN algorithm, LSVM, random trees, SVM, Tree-AS, CHAID, Quest, C&R tree, and neural networks. Among them, logistic regression and Bayesian network had the best overall accuracy for predicting the overall survival (dead vs. alive). The analysis used a custom field (genes) assignment. The target variable was the overall survival as a dichotomic (binary) variable (dead vs. alive). The inputs (predictive genes) were the most relevant genes (*n* = 50) that were previously identified in the Methods 1 (*n* = 19), 2 (*n* = 15), and the MCL35 signature (*n* = 17), as follows: *ADAMDEC1*, *ADGRG2*, *AHR*, *AMOTL2*, *AR*, *ATL1*, *BST2*, *CCNB2*, *CD8B*, *CDC20*, *CDKN3*, *CEACAM6*, *CFB*, *CSF1*, *E2F2*, *ESPL1*, *FABP5*, *FAM83D*, *FMNL3*, *FOXM1*, *GCNA*, *GLIPR1*, *ID1*, *IGFBP7*, *IL6ST*, *ITGAX*, *KCTD12*, *KIF18A*, *KIF2C*, *MKI67*, *NCAPG*, *PALLD*, *PCK2*, *PEMT*, *PIK3CD*, *POGLUT3*, *RAB13*, *RGS1*, *ROBO4*, *RPGRIP1L*, *RRAS*, *SELENOP*, *TAMM41*, *TMEM176B*, *TOP2A*, *TYMS*, *YBX3*, *ZCCHC4*, *ZDHHC21*, and *ZWINT.* A total of 13 models were selected and ranked according to their overall accuracy for predicting the overall survival. In the modeling, every possible combination of options was tested, and the best models were saved. Of note, in the final models not all the genes were necessary or contributed to the model, and only the best combinations were selected (e.g., 50 genes in the Bayesian network but only 6 in the CHAID tree).

**Table 5:** Function and association of the highlighted genes in neoplasia.

Gene	Function	Role in Cancer
*KIF18A*	Microtubule motor activity, role in mitosis	Overexpressed in various types of cancer; inhibitors are available [73]
*YBX3*	Translation repression, negative regulation of intrinsic apoptosis signaling	Related to myelodysplastic syndromes and acute myeloid leukemia [74]
*PEMT*	Negative regulation of cell proliferation, positive regulation of lipoprotein metabolic process	Critical role in breast cancer progression [75]
*GCNA*	Acidic repeat-containing protein, expressed in germ cells (testis)	Regulate genome stability [76,77]
*POGLUT3*	Protein glucosyltransferase, specifically targets extracellular EGF repeats of proteins (NOTCH1 and NOTCH3)	Related to glioblastoma multiforme tumorigenesis [78]
*SELENOP*	Transport of selenium, response to oxidative stress	Prostate cancer recurrence [79]
*AMOTL2*	Actin cytoskeleton organization, angiogenesis, cell migration, Wnt-signaling pathway	Angiogenesis in pancreatic, and proliferation in lung cancer [80,81]
*IGFBP7*	Cell adhesion, metabolic process (retinoic acid, cortisol), regulation of cell growth	Prognosis of acute lymphoblastic leukemia [82]
*KCTD12*	GABA-B receptors auxiliary subunit	Proliferation in breast cancer [83]
*ADGRG2*	G protein-coupled receptor signaling pathway	Tumor suppressor in endometrial cancer [84]
*TYMS*	Regulation of mitotic cell cycle (G1/S transition)	Association with non-Hodgkin lymphomas, prognosis of pancreatic cancer [85,86]

The gene information is based on UniProt [54], and Genecards [55]. *TYMs* was highlighted in Method 2; the rest of genes in Method 1.

## Data Availability

The gene expression data (GEO data sets) were obtained from the publicly available database of the NCBI resources webpage, located at https://www.ncbi.nlm.nih.gov/gds (accessed on 15 August 2021).

## Appendix A

**Table A1:** Multilayer Perceptron Neural Network Analysis of Mantle Cell Lymphoma (Method 1).

Gene	Num. Genes Top 70%	Case Processing Summary				Network Layers					Model Summary					Classification						Area under the Curve (AUC)
		Training		Testing		Input	Hidden		Output		Training			Testing		Training (% Correct)			Testing (% Correct)			
		Num.	%	Num.	%	Units	Num.	Units	Num.	Units	Cross Entropy Error	Incorrect Predictions %	Training Time	Cross Entropy Error	Incorrect Predictions %	Observed 0	Observed 1	Overall	Observed 0	Observed 1	Overall	
Dead/Alive	80	84	68.3	39	31.7	20863	1	6	1	2	38.2	21.4	01:04.9	10.4	12.8	67.6	86	78.6	88.9	86.7	87.2	0.90
*SYNE1*	6	90	73.2	33	26.8	20862	1	12	1	2	38.5	18.9	01:05.8	8.8	9.1	59.3	90.5	81.1	66.7	96.3	90.9	0.86
*DAZAP1*	80	87	70.7	36	29.3	20862	1	11	1	2	32.0	14.9	01:06.3	6.4	5.6	64	93.5	85.1	83.3	96.7	94.4	0.92
*MYCN*	154	85	69.1	38	30.9	20862	1	8	1	2	37.5	27.1	01:01.5	14.4	13.2	36.4	85.7	72.9	66.7	93.1	86.8	0.82
*CXCL12*	56	87	70.7	36	29.3	20862	1	8	1	2	40.5	19.5	00:57.4	10.1	8.3	44	95.2	80.5	83.3	93.3	91.7	0.83
*NOTCH2*	20	84	68.3	39	31.7	20862	1	9	1	2	29.9	20.2	00:58.2	11.8	17.9	92.3	36.8	79.8	93.1	50	82.1	0.90
*CDK4*	47	87	70.7	36	29.3	20862	1	11	1	2	30.4	13.8	00:51.2	13.8	22.2	91.3	66.7	86.2	100	27.3	77.8	0.89
*BMI1*	25	93	85.6	30	24.4	20862	1	8	1	2	53.0	26.9	00:56.3	13.2	16.7	71.7	74.5	73.1	93.8	71.4	83.3	0.81
*ING1*	94	76	61.8	47	38.2	20862	1	10	1	2	36.3	17.1	00:52.7	22.7	27.7	50	93.1	82.9	30.8	88.2	72.3	0.76
*NSD2*	38	91	74	32	26	20862	1	9	1	2	43.0	20.9	01:04.7	15.1	15.6	82.4	75	79.1	91.7	80	84.4	0.86
*PTK2*	6	93	75.6	30	24.4	20862	1	13	1	2	40.2	16.1	01:07.3	7.9	10	97.1	43.5	83.9	91.3	85.7	90	0.85
*PIK3CA*	4	76	61.8	47	38.2	20862	1	10	1	2	26.4	13.2	00:52.4	17.7	12.8	94.8	61.1	86.8	94.3	66.7	87.2	0.88
*CHEK1*	86	91	74	32	26	20862	1	9	1	2	45.3	27.5	00:58.7	12.9	18.8	68.8	76.7	72.5	92.9	72.2	81.3	0.85
*CHEK2*	8	90	73.2	33	26.8	20862	1	10	1	2	39.8	18.9	01:07.6	13.0	15.2	77.3	84.8	81.1	83.3	86.7	84.8	0.88
*PIK3CD*	50	82	66.7	41	33.3	20862	1	10	1	2	17.6	11.0	01:08.1	14.6	14.6	90.9	86.8	89	90.9	78.9	85.4	0.96
*XIAP*	22	85	69.1	38	30.9	20862	1	12	1	2	40.2	18.8	00:49.9	17.7	23.7	83.7	78.6	81.2	85.7	64.7	76.3	0.87
*PAX5*	23	88	71.5	35	28.5	20862	1	7	1	2	45.3	27.3	00:55.2	13.0	8.6	20	93.7	72.7	50	100	91.4	0.75
*BCL2L11*	12	71	57.7	52	42.3	20862	1	5	1	2	29.9	19.7	00:50.1	24.2	23.1	92.6	41.2	80.3	94.9	23.1	76.9	0.82
*BORCS8_MEF2B*	12	85	69.1	38	30.9	20862	1	11	1	2	39.2	21.2	00:53.3	11.6	10.5	40.9	92.1	78.8	55.6	100	89.5	0.83
*PTEN*	86	84	68.3	39	31.7	20862	1	10	1	2	36.0	20.2	00:57.0	12.2	7.7	92.1	42.9	79.8	93.3	88.9	92.3	0.85
*MYC*	10	84	68.3	39	31.7	20862	1	9	1	2	28.9	16.7	00:56.2	14.2	20.5	87.7	68.4	83.3	96.4	36.4	79.5	0.90
*CCND1*	23	87	70.7	36	29.3	20862	1	8	1	2	38.3	23.0	01:03.5	6.7	2.8	92.3	31.8	77	96.4	100	97.2	0.89
*MKI67*	2	93	75.6	30	24.4	20862	1	10	1	2	40.2	20.4	01:04.6	11.7	16.7	78	81.4	79.6	85.7	81.3	83.3	0.89
*CCND2*	46	76	61.8	47	38.2	20862	1	9	1	2	32.4	21.1	00:54.9	17.7	14.9	90.7	50	78.9	92.3	50	85.1	0.84
*CDKN2A*	112	91	74	32	26	20862	1	14	1	2	22.0	9.9	00:53.6	11.3	21.9	94.4	73.7	90.1	91.3	44.4	78.1	0.93
*CDKN2C*	6	90	73.2	33	26.8	20862	1	8	1	2	46.7	26.7	00:58.1	13.5	15.2	67.4	78.7	73.3	89.5	78.6	84.8	0.85
*TERT*	205	82	66.7	41	33.3	20862	1	9	1	2	34.6	20.7	01:00.8	14.9	19.5	93.7	31.6	79.3	93.3	45.5	80.5	0.85
*NOTCH1*	15	85	69.1	38	30.9	20862	1	11	1	2	32.4	17.6	00:49.1	16.3	21.1	88.2	58.8	82.4	88.5	58.3	78.9	0.85
*RB1*	47	88	71.5	35	28.5	20862	1	12	1	2	48.9	27.3	00:56.3	14.3	17.1	65.1	80	72.7	78.9	87.5	82.9	0.83
*Combined*	18	91	74	32	26	20835	1	8	29	58	1348.9	25.7	01:22.2	525.3	29.4	-	-	74.3	-	-	70.6	-
Average		85.9	70.1	37.1	30.2	20861	1	9.6	-	-	80.4	20.1	-	30.6	15.8	75.0	70.8	79.9	84.2	73.5	84.2	0.9

Input layer: standardized rescaling method for covariates. Hidden layer: hyperbolic tangent activation function. Output layer: softmax activation function, cross-entropy error function. Model summary, training, one consecutive step(s) with no decrease in error (error computations are based on the testing sample) as stopping rule.

**Table A2:** Radial Basis Function Neural Network Analysis of Mantle Cell Lymphoma (Method 1).

Gene	Num. Genes Top 70%	Case Processing Summary				Network Layers					Model Summary					Classification						Area under the Curve (AUC)
		Training		Testing		Input	Hidden		Output		Training			Testing		Training (% Correct)			Testing (% Correct)			
		Num.	%	Num.	%	Units	Num.	Units	Num.	Units	Sum of Squares Error	Incorrect Predictions %	Training Time	Sum of Squares Error	Incorrect Predictions %	Observed 0	Observed 1	Overall	Observed 0	Observed 1	Overall %	
Dead/Alive	37	92	74.8	31	25.2	20863	1	8	1	2	16.9	27.2	04:13.3	6.7	38.7	45.5	88.1	72.8	10.0	85.7	61.3	0.73
*SYNE1*	18	85	69.1	38	30.9	20862	1	8	1	2	10.4	17.6	02:46.3	7.4	23.7	40.9	96.8	82.4	27.3	96.3	76.3	0.79
*DAZAP1*	28	80	65	43	35	20862	1	6	1	2	8.2	16.3	02:24.1	3.1	9.3	81.8	84.5	83.8	100.0	88.2	90.7	0.93
*MYCN*	48	82	66.7	41	33.3	20862	1	6	1	2	11.1	20.7	02:32.2	7.4	31.7	30.0	95.2	79.3	9.1	90.0	68.3	0.78
*CXCL12*	50	82	66.7	41	33.3	20862	1	5	1	2	12.7	22.0	02:39.9	8.2	26.8	10.0	100.0	78.0	0.0	100.0	73.2	0.74
*NOTCH2*	29	92	74.8	31	25.2	20862	1	10	1	2	11.7	15.2	03:18.6	4.9	25.8	98.6	35.0	84.8	100.0	11.1	74.2	0.80
*CDK4*	16	82	66.7	41	33.3	20862	1	10	1	2	11.4	20.7	02:21.8	4.9	17.1	98.3	27.3	79.3	100.0	0.0	82.9	0.83
*BMI1*	41	90	73.2	33	26.8	20862	1	5	1	2	20.0	34.4	03:21.6	7.4	39.4	77.6	51.2	65.6	100.0	35.0	60.6	0.70
*ING1*	40	79	64.2	44	35.8	20862	1	4	1	2	14.8	26.6	02:14.7	7.6	22.7	0.0	100.0	73.4	0.0	100.0	77.3	0.60
*NSD2*	39	92	74.8	31	25.2	20862	1	10	1	2	13.6	20.7	03:11.6	4.1	9.7	85.7	72.1	79.3	85.7	94.1	90.3	0.88
*PTK2*	19	90	73.2	33	26.8	20862	1	3	1	2	16.2	24.4	03:15.7	5.8	24.2	100.0	0.0	75.6	100.0	0.0	75.8	0.64
*PIK3CA*	46	79	64.2	44	35.8	20862	1	8	1	2	12.5	24.1	02:23.1	7.7	25.0	93.3	21.1	75.9	100.0	0.0	75.0	0.74
*CHEK1*	51	92	74.8	31	25.2	20862	1	8	1	2	16.4	26.1	03:12.5	7.0	41.9	78.6	70.0	73.9	50.0	72.7	58.1	0.80
*CHEK2*	80	88	71.5	35	28.5	20862	1	9	1	2	13.5	25.0	02:57.1	5.9	22.9	59.1	90.9	75.0	66.7	88.2	77.1	0.86
*PIK3CD*	47	79	64.2	44	35.8	20862	1	3	1	2	12.1	20.3	02:15.3	8.0	27.3	66.7	90.7	79.7	63.3	92.9	72.9	0.83
*XIAP*	89	79	64.2	44	35.8	20862	1	8	1	2	10.7	17.7	02:20.4	11.0	43.2	88.4	75.0	82.3	66.7	47.8	56.8	0.80
*PAX5*	81	89	72.4	34	27.6	20862	1	9	1	2	14.5	24.7	02:55.3	6.0	26.5	13.0	97.0	75.3	0.0	96.2	73.5	0.71
*BCL2L11*	28	88	71.5	35	28.5	20862	1	8	1	2	10.9	14.8	02:51.2	4.1	14.3	100.0	43.5	85.2	96.4	42.9	85.7	0.86
*BORCS8_MEF2B*	41	86	69.9	37	30.1	20862	1	3	1	2	13.8	23.3	02:45.9	5.8	18.9	19.0	95.4	76.7	30.0	100.0	81.1	0.76
*PTEN*	23	92	74.8	31	25.2	20862	1	7	1	2	11.1	16.3	03:14.2	3.5	12.9	95.4	55.6	83.7	92.9	33.3	87.1	0.84
*MYC*	18	92	74.8	31	25.2	20862	1	9	1	2	9.8	16.3	03:31.2	4.1	25.8	91.8	52.6	83.7	95.0	36.4	74.2	0.90
*CCND1*	42	82	66.7	41	33.3	20862	1	10	1	2	11.2	19.5	02:29.4	6.0	26.8	88.3	59.1	80.5	87.9	12.5	73.2	0.81
*MKI67*	37	90	73.2	33	26.8	20862	1	10	1	2	12.6	21.1	03:00.8	5.0	21.2	88.0	67.5	78.9	78.6	78.9	78.8	0.89
*CCND2*	40	79	64.2	44	35.8	20862	1	4	1	2	12.3	24.1	02:14.5	7.6	25.0	100.0	0.0	75.9	100.0	0.0	75.0	0.74
*CDKN2A*	56	92	74.8	31	25.2	20862	1	6	1	2	14.1	20.7	03:02.7	5.0	25.8	97.2	15.0	79.3	100.0	0.0	74.2	0.73
*CDKN2C*	34	88	71.5	35	28.5	20862	1	9	1	2	17.6	21.6	02:50.9	8.9	34.3	86.8	72.0	78.4	58.3	81.8	65.7	0.78
*TERT*	58	79	64.2	44	35.8	20862	1	10	1	2	10.3	17.7	02:17.2	10.0	27.3	93.7	37.5	82.3	100.0	14.3	72.7	0.71
*NOTCH1*	71	79	64.2	44	35.8	20862	1	3	1	2	12.4	22.8	02:14.6	7.3	25.0	100.0	0.0	77.2	100.0	0.0	75.0	0.74
*RB1*	87	89	72.4	34	27.6	20862	1	2	1	2	22.2	47.2	02:55.3	8.7	55.9	100.0	0.0	52.8	100.0	0.0	44.1	0.49
*Combined*	87	93	75.6	30	24.4	20835	1	14	29	58	366.4	20.4	09:53.4	147.2	23.7	-	-	79.6	-	-	76.3	-
Average		86.0	69.9	37.0	30.1	20861	1	7.2			25.0	22.3		11.2	26.4	73.4	58.4	77.7	69.6	51.7	73.6	0.77

Input layer: standardized rescaling method for covariates. Hidden layer: softmax activation function. Output layer: identity activation function, sum of squares error function. Model summary, testing, sum of square error (the number of hidden units is determined by the testing data criterion: The “best” number of hidden units is the one that yields the smallest error in the testing data).

**Table A3:** Multivariate Cox regression analysis for predicting overall survival outcome (Method 1).

Num	Gene	B	SE	Wald	df	*p* Value	Hazard Risk	95.0% CI for HR	
								Lower	Upper
1	*KIF18A*	2.7	0.3	58.3	1	<0.001	14.2	7.2	28.1
2	*YBX3*	0.8	0.2	19.0	1	<0.001	2.2	1.6	3.2
3	*GCNA*	0.9	0.2	14.6	1	<0.001	2.5	1.6	4.1
4	*POGLUT3*	1.2	0.3	13.4	1	<0.001	3.2	1.7	6.0
5	*AMOTL2*	0.9	0.3	10.1	1	0.001	2.5	1.4	4.3
6	*RAB13*	1.2	0.4	9.8	1	0.002	3.3	1.6	7.0
7	*ZCCHC4*	1.1	0.3	9.5	1	0.002	2.9	1.5	5.7
8	*PEMT*	0.6	0.2	8.4	1	0.004	1.9	1.2	2.8
9	*RRAS*	0.8	0.4	4.7	1	0.029	2.2	1.1	4.4
10	*PALLD*	0.6	0.3	3.9	1	0.048	1.8	1.0	3.1
11	*ADAMDEC1*	0.7	0.4	3.5	1	0.063	1.9	1.0	3.9
12	*ADGRG2*	0.4	0.2	2.8	1	0.094	1.5	0.9	2.3
13	*IGFBP7*	−1.5	0.3	20.3	1	<0.001	0.2	0.1	0.4
14	*TMEM176B*	−1.6	0.4	18.9	1	<0.001	0.2	0.1	0.4
15	*SELENOP*	−1.0	0.2	15.6	1	<0.001	0.4	0.2	0.6
16	*RPGRIP1L*	−0.5	0.1	10.5	1	0.001	0.6	0.5	0.8
17	*TAMM41*	−0.8	0.3	7.5	1	0.006	0.4	0.3	0.8
18	*KCTD12*	−1.2	0.5	7.5	1	0.006	0.3	0.1	0.7
19	*RGS1*	−0.4	0.2	4.5	1	0.034	0.7	0.5	1.0

Cox regression, backward conditional.

**Table A4:** Multivariate Cox regression overall survival analysis between MKI67 and the 10 highlighted genes (Method 1).

Gene	B	SE	Wald	df	Sig.	HR	95.0% CI for HR	
							Lower	Upper
*MKI67*	1.3	0.3	20.5	1	0.000	3.8	2.1	6.8
*YBX3*	0.9	0.3	11.3	1	0.001	2.6	1.5	4.4
*SELENOP*	−0.5	0.3	3.0	1	0.085	0.6	0.3	1.1
*POGLUT3*	0.6	0.2	6.9	1	0.009	1.9	1.2	3.1
*ADGRG2*	−0.7	0.3	4.5	1	0.035	0.5	0.2	0.9
*GCNA*	0.8	0.3	5.3	1	0.021	2.2	1.1	4.2
*KIF18A*	1.5	0.3	26.6	1	0.000	4.3	2.5	7.6
*PEMT*	0.8	0.3	6.6	1	0.010	2.1	1.2	3.8

Multivariate Cox regression analysis, backward conditional. HR, hazard risk. Note: There are only 8 genes because it is a multivariate Cox regression analysis with the backward conditional method. In this method, the nonsignificant variables are eliminated.

**Table A5:** Multilayer perceptron analysis of the immuno-oncology pathways (Method 2).

Pathway	Num. Genes Top 70%	Case Processing Summary				Network Layers					Model Summary					Classification						Area under the Curve (AUC)
		Training		Testing		Input	Hidden		Output		Training			Testing		Training (% Correct)			Testing (% Correct)			
		Num.	%	Num.	%	Units	Num.	Units	Num.	Units	Cross Entropy Error	Incorrect Predictions %	Training Time	Cross Entropy Error	Incorrect Predictions %	Observed Alive	Observed Dead	Overall	Observed Alive	Observed Dead	Overall %	
Cancer Transcriptome	13	84	68.3	39	31.7	1785	1	6	1	2	41.1	27.4	00:03.9	17.6	23.1	58.8	82.0	72.6	55.6	83.3	76.9	0.84
Pan Cancer Human IO360	15	84	68.3	39	31.7	727	1	8	1	2	22.5	13.1	00:01.4	14.7	15.4	82.4	90.0	86.9	88.9	83.3	84.6	0.94
Pan Cancer Immune Profiling	1	84	68.3	39	31.7	707	1	5	1	2	44.9	26.2	00:01.5	15.0	12.8	64.7	80.0	73.8	88.9	86.7	87.2	0.82
Pan Cancer Progression	18	84	68.3	39	31.7	715	1	11	1	2	51.2	32.1	00:01.7	18.7	12.8	29.4	94.0	67.9	66.7	93.3	87.2	0.74
Pan Cancer Pathways	6	84	68.3	39	31.7	712	1	8	1	2	36.9	21.4	00:01.8	16.8	15.4	67.6	86.0	78.6	77.8	86.7	84.6	0.89
Metabolic Pathways	27	84	68.3	39	31.7	737	1	14	1	2	39.8	22.6	00:01.6	13.7	17.9	55.9	92.0	77.4	66.7	86.7	82.1	0.87
Immune Exhaustion	12	84	68.3	39	31.7	720	1	10	1	2	47.2	31.0	00:01.6	18.2	17.9	50.0	82.0	69.0	66.7	86.7	82.1	0.79
Human Inflammation	23	84	68.3	39	31.7	247	1	9	1	2	33.7	17.9	00:00.6	16.6	23.1	73.5	88.0	82.1	55.6	83.3	76.9	0.89
Host Response	8	84	68.3	39	31.7	747	1	9	1	2	41.1	21.4	00:01.6	18.1	20.5	67.6	86.0	78.6	66.7	83.3	79.5	0.83
Autoimmune	13	84	68.3	39	31.7	719	1	10	1	2	11.9	6.0	00:01.5	12.5	10.3	88.2	98.0	94.0	88.9	90.0	89.7	0.98
Organ Transplantation	12	84	68.3	39	31.7	728	1	11	1	2	41.5	21.4	00:01.6	15.7	10.3	64.7	88.0	78.6	88.9	90.0	89.7	0.85

Input layer: standardized rescaling method for covariates. Hidden layer: hyperbolic tangent activation function. Output layer: softmax activation function, cross-entropy error function. Model summary, training, one consecutive step(s) with no decrease in error (error computations are based on the testing sample) as stopping rule.

**Table A6:** Overall survival of the pan cancer series using the risk-scores.

Subtype	Overall	Low-Risk	High-Risk	K–M Log-Rank *p* Value	Cox *p* Value	Cox HR	95% CI for HR	
							Lower	Higher
Breast	962	821	141	4.0 × 10^−17^	6.5 × 10^−15^	4.0	2.8	5.6
Lung	475	426	49	1.0 × 10^−10^	1.1 × 10^−9^	3.3	2.3	4.9
Prostate	497	446	51	1.5 × 10^−4^	2.0 × 10^−3^	9.2	2.3	37.2
Colorectal	466	415	51	1.4 × 10^−5^	3.3 × 10^−5^	2.9	1.7	4.8
Cervix	191	169	22	3.4 × 10^−10^	8.9 × 10^−8^	7.7	3.6	16.2
Stomach	440	293	147	2.6 × 10^−4^	3.1 × 10^−4^	1.8	1.3	2.4
Skin (melanoma)	335	177	158	3.2 × 10^−10^	1.3 × 10^−9^	2.6	1.9	3.5
Bladder	389	207	182	9.2 × 10^−13^	9.7 × 10^−12^	3.0	2.2	4.1
Ovary	247	217	30	0.6 × 10^−5^	1.5 × 10^−5^	2.9	1.8	4.6
DLBCL	414	289	125	3.3 × 10^−16^	1.5 × 10^−14^	3.3	2.5	4.5
Kidney	792	470	322	5.9 × 10^−17^	2.5 × 10^−15^	3.2	2.4	4.3
Uterus (endometrium)	247	214	33	5.5 × 10^−11^	2.4 × 10^−8^	7.4	3.7	15.0
Leukemia (AML)	149	115	34	1.9 × 10^−14^	7.0 × 10^−12^	5.5	3.4	9.0
Pancreas	176	109	67	0.4 × 10^−5^	9.0 × 10^−6^	2.6	1.7	3.9
Thyroid	489	434	55	9.9 × 10^−12^	6.4 × 10^−7^	17.4	5.6	53.5
Liver	361	197	164	6.7 × 10^−10^	4.0 × 10^−9^	3.0	2.1	4.3
CNS (GBM)	659	209	450	2.6 × 10^−17^	8.9 × 10^−15^	4.5	3.1	6.6
Overall	7289	5208	2081	2.8 × 10^−178^	2.5 × 10^−159^	3.3	2.9	3.6

K–M, Kapan–Meier; HR, hazard risk, DLBCL, diffuse large B-cell lymphoma; AML, acute myeloid leukemia; CNS, central nervous system; GBM, glioblastoma multiforme. This analysis is univariate.
